# Thermal, Mechanical, and Barrier Properties of PHBV Nanocomposites via TiO_2_ Incorporation for Sustainable Food Packaging

**DOI:** 10.3390/polym18010011

**Published:** 2025-12-19

**Authors:** Karlo Grgurević, Martina Miloloža Nikolić, Dajana Kučić Grgić, Vesna Ocelić Bulatović

**Affiliations:** Faculty of Chemical Engineering and Technology, University of Zagreb, Trg Marka Marulića 19, 10000 Zagreb, Croatia; kgrgurevi@fkit.unizg.hr (K.G.); miloloza@fkit.unizg.hr (M.M.N.); dkucic@fkit.unizg.hr (D.K.G.)

**Keywords:** poly(3-hydroxybutyrate-*co*-3-hydroxyvalerate), titanium dioxide, nanocomposite films, thermal, mechanical, barrier properties, morphology

## Abstract

Poly(3-hydroxybutyrate-co-3-hydroxyvalerate) (PHBV) is a biodegradable polyester considered for food packaging, though its mechanical and barrier limitations pose challenges. This study assessed PHBV/TiO_2_ nanocomposites for packaging applications. Differential scanning calorimetry revealed reduced crystallinity and lower melting points with an increase in TiO_2_ content. Thermal stability improved at 1% and 3% TiO_2_, raising onset temperatures to 283 °C and 284 °C, respectively. Scanning electron microscopy and FTIR confirmed uniform nanoparticle dispersion without agglomeration. Tensile tests showed decreasing strength and modulus from 1% to 7% TiO_2_, with peak elongation at 3%, whereas viscosity behavior declined with higher nanoparticle loading. Low portions of nanoparticles (1% and 3%) induced the improvement in barrier properties against oxygen and water vapor. The highest biodegradation rate occurred at 7% TiO_2_. Overall, the nanocomposites’ properties tend to deteriorate with the addition of higher portions of TiO_2_. Thus, despite some improvements, the nanocomposites did not deliver consistent, multi-property enhancements to justify use in food packaging. Key metrics like sealability and appearance were not evaluated. Future research should explore surface-treated TiO_2_, alternative fillers, compatibilizers, and optimized processing, alongside standardized safety assessments for food-contact applications.

## 1. Introduction

Plastics have enabled hygienic, durable, and low-cost food packaging for decades, but their persistence in the environment has raised legitimate concerns. In response, the European Union has enacted a series of measures (e.g., single-use restrictions, higher recycling targets). However, these policy steps have had limited leverage on global marine litter, as most mismanaged plastic that enters the oceans originates outside Europe—predominantly in parts of Asia and Africa—while Europe’s direct share is comparatively small. Consequently, a complementary route is materials innovation: developing biodegradable, bio-sourced alternatives that can meet packaging performance at an acceptable cost. Conventional petroleum-based materials were and are massively used for food packaging applications, due to their low cost, durability, transparency, as well as good mechanical performance and good barrier properties against oxygen, water vapor and carbon dioxide. However, they proved not to be biodegradable in a short period of time and tend to accumulate in the environment. In this manner, green materials, i.e., environmentally friendly polymers, are intensively investigated as alternatives to the mentioned conventional plastics. Additionally, the green polymers are produced from natural biomass sources, thus satisfying the needs of the circular economy. The production cost of biodegradable food packaging is influenced by several factors, including the choice of feedstock, production scale, and process optimization. While initial production costs may be higher compared to conventional plastics, advancements in the use of renewable feedstocks are expected to reduce costs over time. For instance, utilizing waste-based feedstocks can significantly lower material costs, making biodegradable packaging more competitive in the market. Market demand for biodegradable packaging is growing, driven by increasing consumer awareness and regulatory pressures to reduce plastic waste. Biodegradable packaging’s biodegradability, coupled with its mechanical properties suitable for packaging applications, positions it as a promising alternative to traditional plastics. However, in order for these materials to have adequate barrier and mechanical properties similar to those of the petroleum-based polymers, they need to be adjusted with additives, such as plasticizers, compatibilizers, or fillers [1,2,3,4,5].

Polyhydroxyalkanoates (PHAs) are microbially produced, biodegradable polyesters that can be made from second-generation feedstocks (e.g., agro-industrial and food waste), offering a renewable route to packaging materials [6]. The microbes used for this kind of biotechnological production are mainly bacteria and Archaea (mainly Haloarchaea) [7]. Based on the microorganism’s “preference”, the large-scale equipment has to be adjusted, thus creating a large scale of the PHA production costs. Based on the literature information, a price of USD 5.77 per kilogram of the produced PHA can be achieved on the basis of 9000 tons produced annually. This price involves all of the steps in the production process (fermentation, extraction, purification); however, with the continuous development of the steps of the production process, the production costs can be minimized and implemented in various industrial establishments, such as the food industry and wastewater treatment plants [8].

Among all of the PHAs, poly(3-hydroxybutyrate-*co*-3-hydroxyvalerate) (PHBV) is widely studied for food-contact use due to its biocompatibility, UV resistance, and balanced baseline properties [9,10,11]. However, as mentioned before, PHBV’s commercial uptake is limited by production cost and processing constraints, and its various properties, such as inherent brittleness, low elongation, and modest O_2_/H_2_O barrier, restrict performance in demanding shelf-life applications [12,13,14,15]. Against this backdrop, recent summaries of PHBV modification routes make an important distinction: large gains in ductility are typically achieved by changing composition (e.g., higher 3-HV or mcl-PHA segments), by compatibilized blends with softer polyesters, or by plasticization/processing, whereas rigid nanofillers mainly tune stiffness/crystallization rather than elongation. For example, adding 7 wt.% multi-walled carbon nanotubes (MWCNTs) increased PHBV’s Young’s modulus by ~172% and tensile strength by ~88%, which is a clear reinforcement effect without a ductility route; conversely, plasticizers such as PEG or lauric acid can raise flexibility but often trade off strength and thermal resistance [16]. In general, the mechanical properties of PHBV highly depend on the valerate portion in its structure. Schmidt et al. (2024) [17] used PHBV with 12% valerate, which showed lower tensile strength and elongation at break, valuing 22.6 ± 0.6 MPa and 4.4 ± 0.3%, respectively. However, the addition of newly synthesized PHBV nanoparticles lowered those values, which indicates the effect of the fillers on the polymer matrix. The addition of the nanoparticles to the polymer is expected to increase its barrier properties, i.e., to lower the gas transmission rate through the polymer matrix. Thus, Díez-Pascal and Díez-Vicente (2014) [18] determined the gradual increase in the barrier properties of PHBV with the addition of ZnO nanoparticles. In that matter, oxygen permeability reduced itself up to 35% with the addition of 4.0 wt.% nanoparticles, improving the barrier performance of PHBV. However, a larger portion of ZnO induced the worsening of the barrier properties, which is why much emphasis should be given to optimizing the portion of nanoparticles added to the polymer.

PHBV biodegradation typically requires specific composting conditions that are not universally available, further narrowing practical deployment. In the PHBV grade used here (Table 1), baseline properties are ≈39 MPa tensile strength and ≈2% elongation at break with Tm ≈ 170–176 °C; throughout this work, any additive effect is judged against these packaging-relevant thresholds. Finally, because nano-enabled materials can release particles during disintegration/biodegradation, their environmental safety depends on potential nanoparticle release and ecotoxicity, in addition to food-contact migration; these assessments are outside the present scope and are flagged for future work [19]. To address these limitations, inorganic nanofillers have been explored to tune PHBV’s thermal, mechanical, and barrier behavior.

Titanium dioxide (TiO_2_) is among the most widely studied and industrially applied nanomaterials due to its unique combination of properties [20]. TiO_2_ is a white, non-toxic, and chemically stable metal oxide commonly used as a pigment in paints, cosmetics, pharmaceuticals, and food products. It is approved for food contact applications by regulatory agencies, and exhibits high refractive index, photocatalytic activity, and ultraviolet (UV) light shielding capabilities [21,22]. However, TiO_2_ can be photocatalytically active under UV and may accelerate matrix degradation unless surface-treated or appropriately screened. When integrated into polymer matrices such as PHBV, TiO_2_ nanoparticles can act as reinforcing agents that enhance the composite’s performance in several ways. Mechanically, TiO_2_ can improve stiffness and reduce brittleness by restricting polymer chain mobility and promoting stress transfer at the filler–matrix interface [20]. Practically, in packaging, TiO_2_ is most commonly used for whiteness/opacification, and its broader use can be constrained by cost, which may limit deployment beyond coloration. Thermally, it can raise the onset of degradation and melting temperatures, contributing to better heat resistance during processing and end-use [23]. Furthermore, TiO_2_ improves barrier properties by creating a more tortuous path for the diffusion of gases and moisture, thereby extending the shelf life of packaged food products [24]. The nanoparticles also enhance opacity and UV resistance, protecting both the packaging material and its contents from photodegradation [25]. Additionally, TiO_2_ has demonstrated antimicrobial activity under certain conditions, suggesting potential applications in active packaging where microbial growth inhibition is desirable [26].

The present study explores the development and characterization of PHBV-based nanocomposite films reinforced with TiO_2_ nanoparticles. Specifically, it evaluates the effects of increasing TiO_2_ content on key performance metrics, including thermal behavior, surface morphology, water vapor permeability, and mechanical strength. By employing techniques such as DSC, TGA, SEM, Attenuated Total Reflectance–Fourier Transform Infrared Spectroscopy (ATR–FTIR), contact angle measurement, mechanical properties, and water vapor and oxygen transmission rate testing, this work seeks to provide a comprehensive understanding of the structure-property relationships in PHBV/TiO_2_ nanocomposites. In addition, biodegradation was evaluated through 56-day soil burial tests, tracking weight loss and surface changes to assess environmental disintegration outside controlled composting. Morphological and spectroscopic analyses revealed how TiO_2_ content influences degradation behavior. This integrated approach deepens understanding of structure property–degradation relationships in PHBV/TiO_2_ films, aiming to develop biodegradable packaging materials that are both high-performing and environmentally responsive. Additionally, nanoparticle-enabled packaging also requires consideration of migration/toxicology under the EU 1935/2004 [27] framework; such testing is outside the present scope. Likewise, this study does not include heat-seal behavior or optical color/whiteness measurements; therefore, no claims are made on those properties. Accordingly, our objective is to test whether PHBV/TiO_2_, as processed here, delivers consistent, multi-property changes large enough to matter for packaging—rather than to assert enhancement a priori.

## 2. Materials and Methods

### 2.1. Materials

The polymer matrix used was poly(3-hydroxybutyrate-*co*-3-hydroxyvalerate) (PHBV), commercially designated as ENMAT™ Y1000P (Tianan Biologic Materials Co., Ltd., Ningbo, China), provided in pellet form. The properties of the PHBV matrix are shown in Table 1. Titanium dioxide (TiO_2_) nanoparticles (~100 nm) were obtained from Sigma-Aldrich (St. Louis, MO, USA) and used as the nanofiller. Both components were stored at 25 °C and 28% relative humidity prior to processing.

### 2.2. Preparation of PHBV/TiO_2_ Nanocomposites

PHBV and TiO_2_ were mechanically mixed in various weight ratios (1, 3, 5, and 7 wt.% TiO_2_) using a Brabender kneader preheated to 180 °C (Table 2). The components were first manually premixed and then introduced gradually at 20 rpm, increasing to 60 rpm for a total mixing time of 10 min. The resulting melt was cooled, shredded, and molded initially using a hydraulic press, Fontuna (model SRB 140 (EC 320 × 320 NB), Rotterdam, Holland) at 180 °C and a pressure of 20 kPa. Then, pressed samples were water-cooled to ambient temperature under the same pressure on a Dake Model 44-226 (Dake Corporation, Grand Haven, MI, USA) press to form films suitable for characterization. The dimensions of the prepared samples varied depending on the specific type of analysis to be conducted. For evaluating mechanical, thermal, and structural characteristics, samples were shaped into specimens measuring 10 mm × 10 mm × 1 mm. In contrast, for barrier properties and soil biodegradation testing, i.e., monitoring the degradation process, through morphological and spectroscopic assessment, the samples were initially compressed into thin film-like sheets by pressing the granules between two plates with Teflon foil. These sheets had an average thickness of approximately 0.4 mm, which varied slightly depending on the TiO_2_ content. The resulting films were then cut into square pieces measuring 15 mm × 15 mm, and each was weighed using a high-precision analytical balance (model AEAAK45, SAB 125i, Adam Equipment Co. Ltd., Milton Keynes, UK) with an accuracy of 0.00001 g.

### 2.3. Characterization Methods

#### 2.3.1. Differential Scanning Calorimetry (DSC)

Thermal characterization was carried out using a differential scanning calorimeter (DSC), specifically the Mettler Toledo DSC 3+ system, Greifensee, Switzerland. Approximately 10–12 mg of each sample was placed in a sealed aluminum crucible. The DSC analysis comprised a first heating run from 25 °C to 200 °C at 10 °C min^−1^ under nitrogen purge (60 mL min^−1^) to remove previous thermal history, followed by cooling to −50 °C and a second heating to 200 °C at the same rate. Melting enthalpy (Δ*H_m_*) and degree of crystallinity (*χ_c_*) were calculated from the second heating run using a reference enthalpy of fusion for 100% crystalline PHBV (Δ*H_m_°* = 146.0 J g^−1^) [28].

#### 2.3.2. Thermogravimetric Analysis (TGA)

Thermal stability was assessed using a Mettler Toledo TGA/DSC 3+ instrument, Greifensee, Switzerland. For each analysis, 10–12 mg of sample was weighed and placed in a platinum crucible. The samples were heated from ambient temperature to 700 °C at a constant heating rate of 10 °C min^−1^ under a continuous flow of high-purity nitrogen gas (99.99%; flow rate 50 mL min^−1^) to prevent oxidative degradation. The onset temperature of decomposition (*T_onset_*), the temperature at maximum weight loss rate (*T_max_*), and the residual mass at 700 °C were recorded for all samples.

#### 2.3.3. Scanning Electron Microscopy (SEM-EDX)

To investigate the morphology of the prepared samples, including both surface topography and fractured cross-sections, scanning electron microscopy (SEM) was employed. The analyses were conducted using a TESCAN VEGA3 scanning electron microscope (Tescan, Brno, Czech Republic), operated at an accelerating voltage of 20 kV. Before imaging, all samples were mounted on aluminum stubs and sputter-coated with a thin layer of platinum and gold to enhance surface conductivity and prevent charging under the electron beam. Fractured surfaces were prepared by cryogenic fracturing, which helps to preserve the intrinsic morphology of the polymeric structure without introducing thermal artifacts. Both surface morphology and microstructural features were examined to evaluate the dispersion of phases and homogeneity of the material blends. In addition to morphological characterization, elemental composition analysis was performed using energy-dispersive X-ray spectroscopy (EDX) integrated with the SEM system. EDX was particularly useful for identifying and mapping the elemental distribution across the sample surface, which is important in confirming the presence and distribution of inorganic fillers or additives.

#### 2.3.4. Attenuated Total Reflectance—Fourier Transform Infrared Spectroscopy (ATR–FTIR)

Chemical structure and potential interactions between PHBV and TiO_2_ were analyzed using ATR–FTIR spectroscopy, PerkinElmer Spectrum Two, Waltham, MA, USA. Each sample was placed directly on the ATR crystal, and spectra were collected in the range of 4000–650 cm^−1^ at a resolution of 4 cm^−1^ with 32 scans per measurement. For the characterization of initial (unaged) nanocomposite films, characteristic absorption bands corresponding to functional groups in PHBV and TiO_2_ (such as ester carbonyl and Ti–O–Ti vibrations) were identified and compared among different formulations to assess potential interactions and composite structure. To assess the chemical changes during biodegradation, film samples were recovered from soil at specified intervals (7, 14, 21, 42, and 56 days), washed with distilled water, and dried at 40 °C to constant weight. ATR–FTIR spectra were then acquired following the same procedure. Changes in intensity and position of characteristic bands (e.g., C=O stretching, C–O–C stretching) were monitored to evaluate chemical modifications associated with environmental degradation. All spectra were baseline-corrected and normalized prior to analysis, and at least three replicates were measured per condition to ensure reproducibility.

#### 2.3.5. Contact Angle Measurements

Surface wettability was assessed using a DataPhysics OCA 20 contact angle goniometer (DataPhysics Instruments GmbH, Filderstadt, Germany). Static contact angles were measured by dispensing 2 μL droplets of deionized water, formamide, and diiodomethane onto the surface of each film at room temperature. For each sample, at least five measurements were performed at different locations, and the mean value was reported. The surface free energy (SFE) of the films was calculated using the Owens–Wendt–Rabel–Kaelble (OWRK) method, based on the measured contact angles.

#### 2.3.6. Barrier Properties

In order to assess the barrier properties of the prepared nanocomposites, water vapor transmission rate (WVTR) and oxygen transmission rate (OTR) were measured. Measurements were conducted using a C403H OTR/WVTR Testing System (Labthink Instruments Co., Ltd., Jinan, China) and were performed on nanocomposites’ thin films with thickness values of ~100 μm. The testing area was 50 cm^2^. WVTR was measured based on the standard ASTM F1249 [29], which included the measurement conditions of 38.0 °C and relative humidity of 90.0%. OTR measurements were taken based on ASTM D3985 [30] at 23.0 °C and 0.0% relative humidity. Both of the testing methods lasted for 30 min.

#### 2.3.7. Mechanical Testing

Mechanical properties were evaluated using a Zwick AllroundLine 20 kN universal testing machine (ZwickRoell, Ulm, Germany). Film specimens (10 mm × 50 mm) were prepared and clamped with an initial gauge length of 30 mm. Tensile tests were performed at a constant crosshead speed of 10 mm min^−1^ at 25 °C. The tensile strength, elongation at break, and Young’s modulus were determined from stress–strain curves. For each sample type, at least five replicate measurements were carried out, and mean values with standard deviations were reported.

#### 2.3.8. Rheological Measurements

Rheological properties of the nanocomposites were tested through oscillatory shear measurements, which were performed using a Discovery HR30 Hybrid Rheometer (TA Instruments, New Castle, DE, USA). The rheometer was equipped with parallel disks 25 mm in diameter. The diameters of the samples were the same, ca. 1.5 mm thick. All of the measurements were performed at 180 °C, under a fixed strain amplitude of 2%. The angular frequency was continuously decreased from 600 rad s^−1^ to 0.1 rad s^−1^.

#### 2.3.9. Biodegradation in Soil

Biodegradation of nanocomposite films was evaluated via controlled soil burial tests over 56 days, following ISO 1755 [31]. Square specimens (15 mm × 15 mm) were weighed (initial mass m_0_) and buried at a depth of 10 cm in garden soil maintained at 58 ± 2 °C and 60% relative humidity. The soil was inoculated with a 50 mL microbial suspension comprising *Pseudomonas aeruginosa*, *Bacillus subtilis*, *Trichoderma* spp., and *Aspergillus niger*, previously cultured in-house. The suspension was prepared by skimming the grown bacterial and fungal cultures from two Petri dishes of each microorganism, putting ca. 25% of each microorganism into the saline solution. Moisture was sustained by the daily application of deionized water. Each specimen was retrieved at intervals of 7, 14, 21, 42, and 56 days, gently rinsed with distilled water, dried at 40 °C to constant weight, and reweighed (*mₜ*). The extent of degradation was determined by the percentage mass loss, Δ*m* (%), calculated according to the following Equation (1):

(1) $$∆m=\frac{\left(m_{0}-m_{t}\right)}{m_{0}}\times 100\%$$

Post-extraction, the samples were visually inspected under an Olympus BX50 (Tokyo, Japan) light microscope (100×) and subsequently examined with a polarizing microscope (Olympus BX53M, 50×, Tokyo, Japan) to identify surface deterioration, such as cracking or edge erosion. To remove residual organic matter and soil aggregates, specimens were washed with 70% ethanol followed by deionized water, then oven-dried at 50 °C for 2 h. Structural modifications during degradation were assessed via ATR–FTIR spectroscopy (IRTracer-100, Shimadzu, Tokyo, Japan).

## 3. Results

### 3.1. DSC Analysis

Differential Scanning Calorimetry (DSC) analysis was performed to investigate the phase transition behavior of neat PHBV and PHBV/TiO_2_ nanocomposites containing 1, 3, 5, and 7 wt.% TiO_2_. The resulting DSC thermograms provided detailed information on the thermal transitions, including the melting temperature (*T_m_*), enthalpy of fusion (Δ*H_m_*), crystallization temperature (*T_c_*), crystallization enthalpy (Δ*H_c_*), and the calculated degree of crystallinity of PHBV (*χ_c_*).

**Figure 1:**
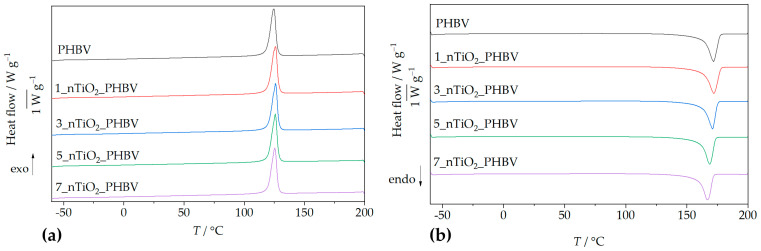
DSC thermograms: (**a**) second heating cycle; (**b**) cooling cycle of PHBV/TiO_2_ nanocomposites in comparison with pure PHBV.

Figure 1 presents the DSC thermograms, i.e., heating cycles (Figure 1a) and cooling cycles (Figure 1b), of the neat PHBV matrix. Only the second heating cycles are presented in Figure 1a, and were used for the thermal transitions determination; the first heating cycles are not shown because their purpose was to erase the polymer’s thermal history. Both in the pure PHBV and the nanocomposites’ second heating cycle, a single endothermic peak appears as a melting point indicator. For neat PHBV, it appears at 171.8 °C, while the enthalpy of fusion was determined to be 92.0 J g^−1^. According to literature [32], PHBV can exhibit two distinct melting endotherms, typically at ~158.0 °C and ~172.0 °C, attributed to partial melting and recrystallization processes. However, this phenomenon is more particular when valerate content exceeds 5 wt.% [32]. The one peak observable in curves on Figure 1a indicates highly homogeneous formation of the crystals, which is why the first endothermic peak is non-existent or drastically reduced. The uniformity of the crystal structure of the polymer matrix was achieved by erasing the thermal history in the first heating cycle. Similar results were obtained in research conducted by Montanheiro et al. (2016) [33], where two endothermic peaks were formed during the first heating rate due to different crystal structures present in PHBV, whilst during the second heating rate, the first melting peak was less pronounced.

By analyzing the heating curves obtained for nanocomposites, the 1 and 3 wt.% of TiO_2_ nanoparticles did not change the melting temperature much, whereas 5% and 7% TiO_2_ nanoparticles decreased it by up to ca. 3 °C and 5 °C, respectively. The shift of the melting peaks towards lower temperatures with higher TiO_2_ loadings suggests a reduction in crystalline domain sizes, likely due to disruption of the PHBV crystal lattice by TiO_2_ nanoparticles [34]. The 1_nTiO_2__PHBV nanocomposite exhibited the highest melting temperature and enthalpy (172.0 °C; 95.8 J g^−1^), indicating enhanced crystallinity, improved chain packing, and possible stabilization of the polymer matrix, in comparison with the neat PHBV. Conversely, the 7_nTiO_2__PHBV nanocomposite exhibited the lowest melting temperature and enthalpy of fusion (166.7 °C; 91.8 J g^−1^), indicating a more disrupted and less thermally stable crystalline structure. This suggests that, although the overall degree of crystallinity is higher (67.6%), the crystals formed are thinner, less ordered, or more imperfect, leading to reduced melting stability [35]. All relevant thermal data, including melting and crystallization temperatures and their corresponding enthalpies, are summarized in Table 3.

The glass transition temperature (*T_g_*) was not detectable in either the heating or cooling thermograms of both neat PHBV and its nanocomposites. This may be attributed to the high crystallinity degree of PHBV, which can obscure the subtle change in heat capacity typically associated with *T_g_* [36,37]. Literature values report the *T_g_* of PHBV in the range of −5 °C to 5 °C [38]. However, during the cooling cycle, a sharp exothermic peak was observed at 124.8 °C (Figure 1), corresponding to the crystallization temperature, with an associated enthalpy of crystallization of 87.5 J g^−1^. Although crystallization temperatures in PHBV/TiO_2_ nanocomposites are slightly elevated compared to neat PHBV, the differences are not significant, while crystallization enthalpies generally decrease with increasing TiO_2_ content. This indicates that TiO_2_ alters the crystallization process through heterogeneous nucleation, increasing complexity and reducing efficiency [35]. The calculated crystallinity degree somewhat rises with the addition of greater amounts of nanoparticles, in comparison with pure PHBV (63.0%). The highest crystallinity degree was obtained for nanocomposites with 5 and 7 wt.% of TiO_2_, and it was 67.6% for both of the nanocomposites. A similar increase in crystallinity degree values was observed in research by Carli et al. (2011) [39], in which thermal properties of PHBV/montmorillonite and PHBV/halloysite nanocomposites were assessed. This behavior of the nanocomposites’ crystallinity was explained by the good distribution of nanoparticles in the polymer matrix and strong interaction between the nanoparticles and polymer chains, which enhanced the formation of crystals [39]. In conclusion, changes in *T*_m_ and *χ*_c_ are small and fall within typical DSC uncertainty. Accordingly, they do not widen the practical heat-sealing/processing window nor imply shelf-life gains. In this study, the DSC results are therefore interpreted as processing diagnostics (thermal history, crystallization trends) rather than application-level improvements.

**Figure 2:**
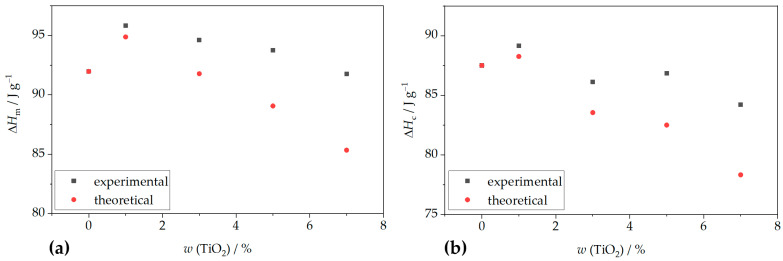
Dependence of TiO_2_ mass portion in PHBV/TiO_2_ nanocomposites on: (**a**) enthalpy of fusion change; (**b**) crystallization enthalpy change.

Nanoparticles influence polymer crystallization behavior through their size, dispersion, and interaction with the matrix, affecting nucleation density, spherulite size, crystallization kinetics, and mechanical properties [40]. To further understand the impact of filler content on crystallization behavior, theoretical values for melting and crystallization enthalpies were calculated. Comparisons with experimental data (Figure 2) show that measured enthalpies are consistently higher than theoretical values, confirming the nucleating effect of TiO_2_ on PHBV crystallization. The nucleation-promoting role of TiO_2_ nanoparticles has also been supported by Venezia et al. [41], who demonstrated that both TiO_2_ and humic substances increase crystallization temperatures by limiting polymer chain mobility and acting as nucleation centers. Similar findings were reported by Metanawin et al. [42] for PBT/TiO_2_ nanocomposites and by Gasmi et al. [43] and Mofokeng et al. [44] for PHBV/PLA/TiO_2_ systems.

### 3.2. TGA Analysis

Thermogravimetric analysis (TGA) was employed to investigate the thermal stability of the neat PHBV matrix and its nanocomposites containing 1, 3, 5, and 7 wt.% TiO_2_. Two characteristic curves were obtained: the thermogravimetric (TG) curve, representing the mass change (%) as a function of temperature, and the derivative thermogravimetric (DTG) curve, which identifies the temperature at which the maximum degradation rate occurs (*T_max_*). The TG curve also provides critical thermal parameters, including the onset degradation temperature (*T_onset_*), the final degradation temperature (*T_f_*), and the residual mass at 700 °C (*R*_700_).

**Figure 3:**
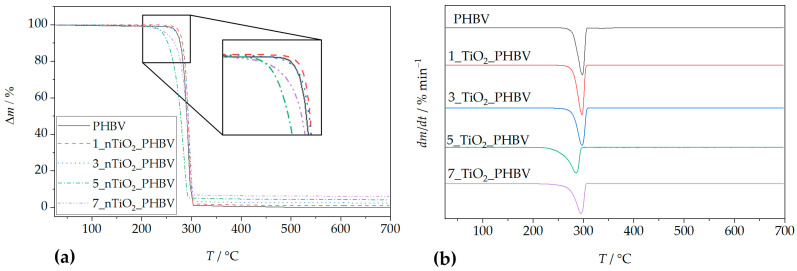
TGA analysis: (**a**) TG curves; (**b**) DTG curves for PHBV/TiO_2_ nanocomposites in comparison with pure PHBV.

The thermal degradation of neat PHBV and all of its nanocomposites proceeds in a single step (Figure 3a). Based on the curve for pure PHBV, the thermal degradation occurs between approximately 265 °C and 310 °C, with a mass loss of 98.7% and a residual mass of 1.3%. The temperature corresponding to the maximum degradation rate (*T_max_*) was observed at 286.6 °C. According to literature sources [45], PHBV undergoes thermal degradation primarily via random scission of ester bonds, following a stereoselective cis-elimination mechanism that produces double bonds along the polymer backbone. Other studies [46] report that degradation also occurs through a β-elimination process involving a six-membered ring transition state. In the early stages of degradation, chain scission leads to the formation of low molecular weight PHBV species and crotonate-terminated chains, followed by the formation of crotonic acid and oligomers. In the final stages, these degradation products, such as carbon dioxide, propylene, acetaldehyde, and other volatile olefinic compounds, further decompose [45]. Some of the volatile olefinic compounds include isopropyl-2-crotonic acid, butyric-2-crotonic acid, β-butyrolactone, and 2-pentenoic acid [47].

Thermal degradation data for the neat PHBV and PHBV/TiO_2_ nanocomposites are summarized in Table 4 and illustrated in Figure 3. The *T_onset_* values of almost all nanocomposites were higher than that of neat PHBV (265.0 °C), indicating improved thermal stability. Specifically, higher *T_onset_* values were 283.0 °C (1 wt.% TiO_2_), 284.0 °C (3 wt.% TiO_2_), and 277.4 °C (7 wt.%). This enhancement is attributed to the barrier effect of TiO_2_ nanoparticles, which impede thermal diffusion and delay degradation in the early stages [32,48]. However, the addition of 5 wt.% TiO_2_ lowered the *T_onset_* value to 260.0 °C. This implicates the occurrence of the threshold effect, meaning that this nanocomposite simply does not follow the trend of the *T_onset_* value change with an increase in TiO_2_ loading, but rather behaves differently, according to the properties of nanoparticles and the polymer matrix independently. The reason for this kind of deviation lies in the disruption of the continuity of the PHBV matrix, i.e., creation of the defect at the interface between the matrix and the filler. These defects act as weak points where thermal degradation can start more easily. The maximum degradation temperatures (*T_max_*) were also elevated in the nanocomposites, except for the 5_nTiO_2__PHBV nanocomposite, which exhibited a lower *T_max_* (285.3 °C) than neat PHBV. This reduction is likely due to structural imperfections in the crystalline regions or the catalytic activity of TiO_2_ [44]. Overall, the presence of TiO_2_ slowed the degradation process by strengthening the interactions between filler and polymer chains, thus delaying the generation and decomposition of volatile degradation products [45]. This property enhances the thermal processability of the material and supports its potential application in food packaging, where increased thermal resistance is advantageous. However, this increased stability may also delay environmental degradation if the material is improperly disposed of. For PHBV film processing (≈170–190 °C), the onset of mass loss (*T_onset_*) remains well above the processing range for all formulations; therefore, the TiO_2_-related shifts in *T_onset_*/*T_max_* observed here do not alter the practical processing safety margin or the heat-seal window. Because these TGA measurements are made under controlled (inert) atmospheres, they are best viewed as diagnostics of comparative thermal stability and residue rather than predictors of ambient storage stability or shelf-life.

Residual mass data in Table 4 reflect the presence of inorganic TiO_2_, which by its nature can not be degraded at temperatures as low as these (700 °C). The 1_nTiO_2__PHBV nanocomposite exhibited the highest mass loss (98.4%), while higher TiO_2_ loadings corresponded to increased residual mass due to the higher inorganic content. These findings are consistent with other studies. For example, Venezia et al. (2023) [41] demonstrated that PHBV/nTiO_2_ nanocomposites containing humic substances degrade in a single step, exhibiting enhanced thermal stability due to an improved polymer network structure. Similar improvements were observed in PHBV nanocomposites reinforced with carbon nanotubes [49], where stronger filler-matrix interactions delayed degradation. In contrast, PHBV/chitosan/ZnO/Ag nanocomposites synthesized by Zare et al. (2019) [46] exhibited a three-step degradation process, attributed to the evaporation of adsorbed water and degradation of chitosan and PHBV. Hussain et al. (2017) [50] reported that hydrogen bonding between cellulose hydroxyl groups and PHBV carbonyl groups enhanced the thermal stability of PHBV/cellulose/ZnO nanocomposites. Aydemir et al. (2016) [51] also reported increased thermal stability in polypropylene/TiO_2_ nanocomposites, attributed to the restricted mobility of the polymer chains. Conversely, PHBV nanocomposites based on organo-modified montmorillonite and halloysite exhibited reduced thermal stability, with *T_max_* shifted to lower temperatures [39]. These results collectively highlight the significant influence of TiO_2_ nanoparticles, and nanoparticles in general, on the thermal degradation behavior of PHBV.

### 3.3. Morphological Analysis of PHBV and PHBV/TiO_2_ Nanocomposites

**Figure 4:**
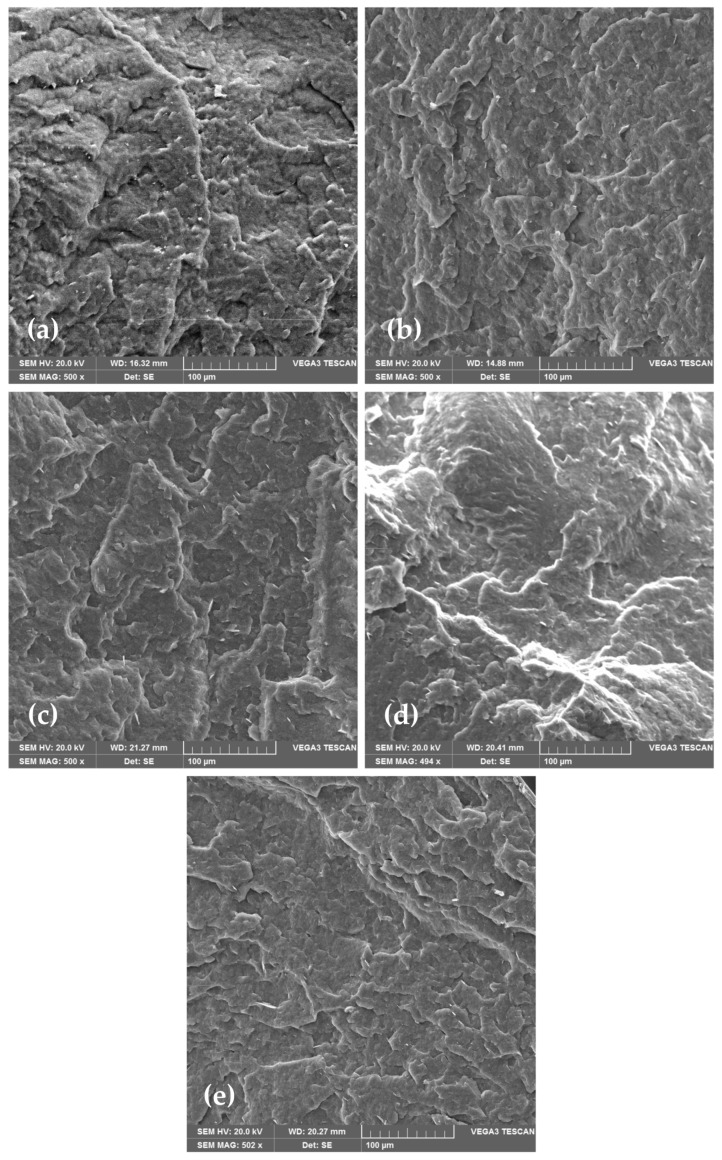
SEM microphotographs of (**a**) pure PHBV; (**b**) 1_nTiO_2__PHBV; (**c**) 3_nTiO_2__PHBV; (**d**) 5_nTiO_2__PHBV; (**e**) 7_nTiO_2__PHBV.

**Figure 5:**
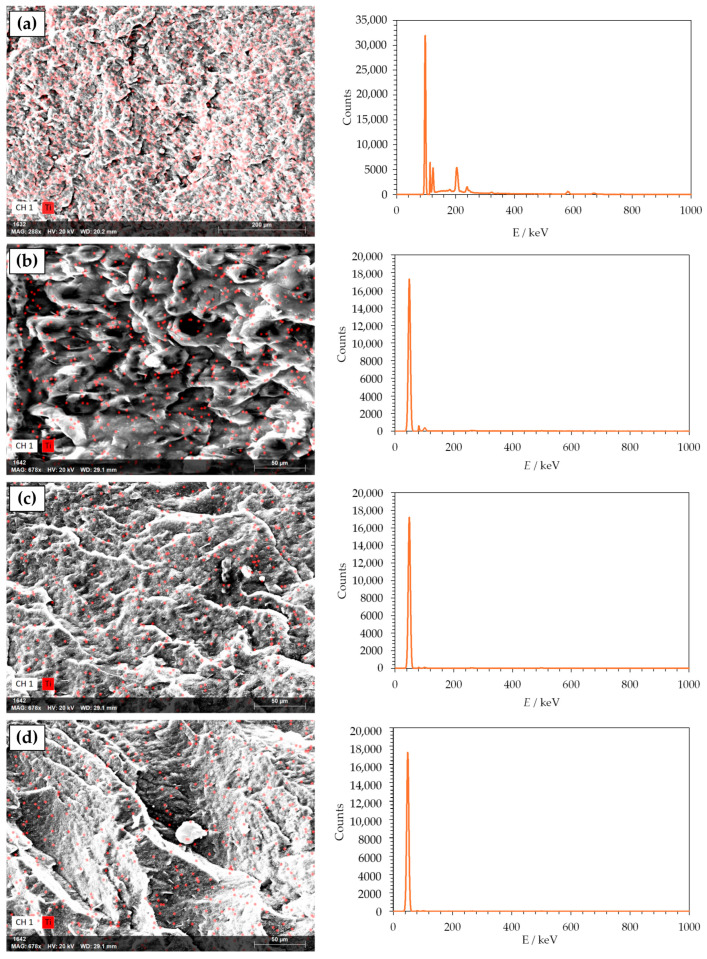
SEM-EDX microphotographs of (**a**) 1_nTiO_2__PHBV; (**b**) 3_nTiO_2__PHBV; (**c**) 5_nTiO_2__PHBV; (**d**) 7_nTiO_2__PHBV.

SEM is an optical technique that provides high-resolution microphotographs of the specific material. Based on the microphotography, a lot of information can be obtained about the material’s morphology. Material’s surface morphology is crucial for the estimation of its properties, such as smoothness, roughness, and porosity, which could influence barrier and mechanical properties of the material. Morphology of PHBV and its nanocomposites was analyzed with a scanning electron microscope (SEM), which is one of the standard methods for microstructure and morphology of the material studies. Figure 4 shows the obtained microphotographs of the neat PHBV matrix and PHBV/TiO_2_ nanocomposites. The PHBV has been shown to have a very dense morphology with a relatively homogeneous and smooth surface. Some irregularities in the surface are the result of the PHBV crystalline structure with a high crystallinity degree of 63.0%, as mentioned before (Table 3) [52]. The microphotographs of nanocomposites show the existence of one phase in every one of the samples, with TiO_2_ nanoparticles dispersed in the polymer matrix. TiO_2_ nanoparticles are seen on the microphotographs as white elongated dots, and they are well dispersed in all of the samples. Nanoparticles did not agglomerate in any of the nanocomposites, which was confirmed with SEM and energy dispersive X-ray spectroscopy (EDX) analysis (Figure 5). SEM-EDX analysis creates a map of the material’s surface, with highlighted spots in different colors, which represent the observable elements, in this case, titanium.

Similar findings were reported by Venezia et al. [31], who investigated PHBV/TiO_2_ nanocomposites incorporating humic substances. The resulting materials exhibited uniform and smooth surfaces, free from visible structural defects. However, at low TiO_2_ loadings, nanoparticle agglomeration was observed, likely due to interactions with the humic substances. Other studies by Bužarovska et al. (2009) [40], Braga et al. (2018) [32], and Mofokeng et al. (2015) [45] also confirmed the same homogeneity of the nanocomposites, i.e., uniformly dispersed TiO_2_ nanoparticles in the polymer matrix.

### 3.4. Nanocomposite Structure

**Figure 6:**
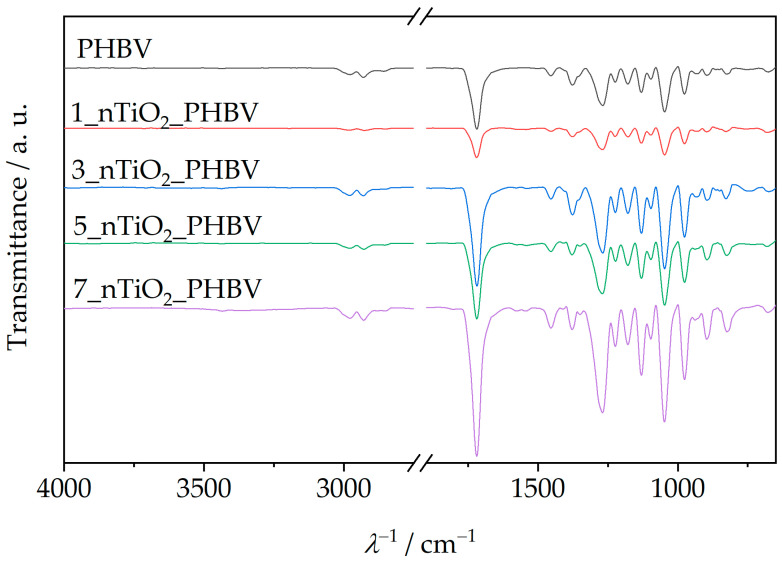
ATR–FTIR spectra of PHBV/TiO_2_ nanocomposites compared to the spectrum of the pure PHBV.

Attenuated total reflectance–Fourier transform infrared spectroscopy (ATR–FTIR) is an analysis method that includes infrared (IR) radiation passing through the sample, thus making the specific chemical bond or functional group in the sample’s structure vibrate. In that way, general information about the chemical structure of the sample can be obtained [51]. ATR–FTIR spectra of the PHBV and all the prepared nanocomposites are shown in Figure 6.

Figure 6 shows the ATR–FTIR spectrum of the neat PHBV, and a few characteristic signals for functional groups can be observed. Absorption peaks at 1252 cm^−1^ and 1275 cm^−1^ represent the saturated ester C–O stretching. Also, characteristic stretching of C–O–C bonds is observable at wavenumber intervals from 1055 cm^−1^ to 1226 cm^−1^. Specifically, a sharp peak at 1181 cm^−1^ represents amorphous C–O–C bonds stretching, while an absorption peak at 1226 cm^−1^ occurs because of crystalline C–O–C bond stretching. Bending of the C–H bond present in methyl, –CH_3_, and methylene group, –CH_2_–, was induced by IR radiation at wavenumbers of 1380 cm^−1^ and 1453 cm^−1^. Peaks present in the wavenumber interval from 2864 cm^−1^ to 2977 cm^−1^ represent stretching of the same bonds. Since PHBV is generally classified as a polyester, its molecule also consists of a characteristic carbonyl functional group, C=O. Due to that fact, the obtained spectrum contains a peak at 1718 cm^−1^, which is intensely sharp because of the molecule’s dipole moment [32,53]. The ATR–FTIR spectra of PHBV/TiO_2_ nanocomposites prepared in this research (Figure 6) show great similarity with the PHBV matrix ATR–FTIR spectrum, explained in the previous paragraph. They show all of the PHBV characteristic signals, with the addition of peaks in the wavenumber interval from 500 cm^−1^ to 800 cm^−1^, which represent Ti–O–Ti bond stretching [54]. Ti–O–Ti bond signals appear at 658 cm^−1^, 678 cm^−1^, 674 cm^−1^, and 677 cm^−1^, on ATR–FTIR spectra for nanocomposites with 1, 3, 5, and 7 wt.%, respectively. All of the specific peaks that appear on the nanocomposites’ ATR–FTIR spectra are shown in Table 5.

### 3.5. Nanocomposite Surface Free Energy

**Figure 7:**
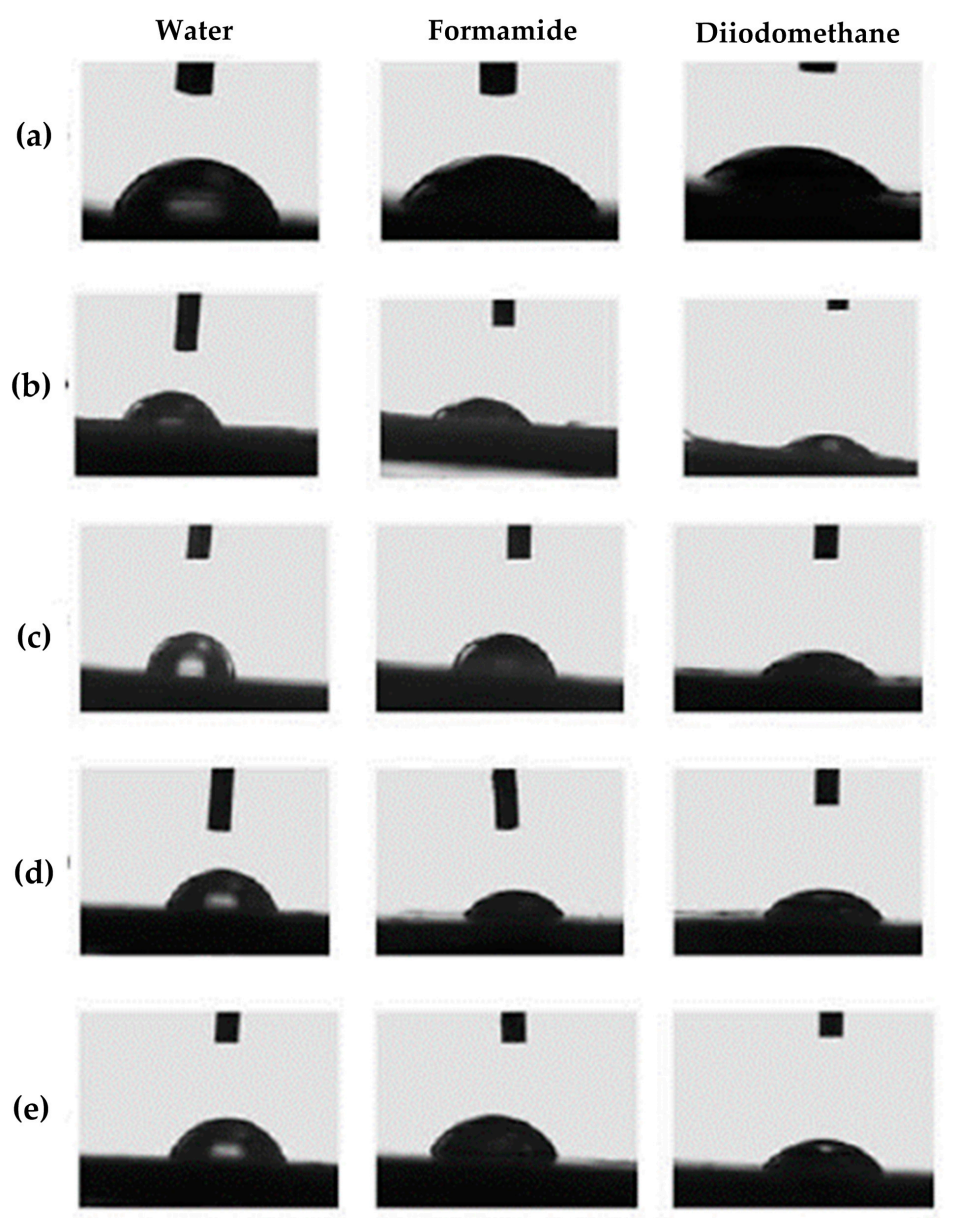
Photographs of the droplets on the surface of (**a**) pure PHBV; (**b**) 1_nTiO_2__PHBV; (**c**) 3_nTiO_2__PHBV; (**d**) 5_nTiO_2__PHBV; (**e**) 7_nTiO_2__PHBV nanocomposites.

In this research, the measurement of the contact angle between specific solvents and nanocomposites has been conducted. The main goal was to obtain the surface free energy values of pure PHBV and PHBV/TiO_2_ nanocomposites. However, since the contact angle measurement provides information about the material’s surface ability to retain moisture, it is a practical method for also determining the applicability of the nanocomposites as a food packaging material. The hydrophilic or hydrophobic nature impacts the interaction of various liquids with the packaging surface, whilst at the same time impacting the adhesion, coating, and cleaning of the surface. Generally speaking, contact angle values lower than 90° indicate that the testing solvents moisturize the surface due to the liquid’s tendency to wet and spread on the surface [55]. Contact angle values also give information about the physical and chemical properties of the materials’ surfaces [55,56]. In direct contact with air, the polymer keeps non-polar saturated groups on the surface layer; however, in contact with a liquid, a favorable reassignment of the polar groups occurs. Interaction of polar segments with polar liquids disables the liquid drop on the surface from shrinking, resulting in the contact angle value being lower than 90° [57]. Table 6 provides the average values of contact angles, while Figure 7 shows the photographs testing the liquid drops’ behavior on the PHBV matrix and nanocomposites’ surfaces.

Contact angle values in this research represent the interaction between the material’s surface and testing liquid, which in this case are water and formamide as polar liquids and diiodomethane as a non-polar liquid. According to Table 6, the contact angle between a water droplet and a pure PHBV surface totals 67.8 ± 1.1°, which means that the surface has a water-moisturizing tendency. In that matter, PHBV can be described as a hydrophilic material. A similar interaction was observed with formamide drops, which resulted in a contact angle value of 54.8 ± 0.4°. The obtained results indicate strong interactions between the PHBV surface and polar liquids. This result might intuitively indicate that contact angles between diiodomethane droplets and the surface of the material should be larger. However, the contact angle value for diiodomethane measured was 41.1 ± 1.2°, which indicates the partial non-polar nature of the PHBV surface. A similar observation was obtained as a result of measuring the contact angle of PHBV in research conducted by Snowdon et al. (2017) [58]. Contact angles of water and diiodomethane on the PHBV surface were measured to be 67.46 ± 0.29° and 46.34 ± 0.033°, respectively, indicating the presence of polar and non-polar regions along the PHBV polymer chains.

Based on the results in Table 6, PHBV/TiO_2_ nanocomposites with various TiO_2_ portions also moisten well with both polar and non-polar liquids. All of the contact angle values of water drops and the nanocomposite surface are lower than 90°, confirming the hydrophilic nature of the nanocomposites. Nanocomposites 1_nTiO_2__PHBV, 5_nTiO_2__PHBV, and 7_nTiO_2__PHBV are furthermore less hydrophilic than the pure PHBV, based on higher contact angle values. However, the 3_nTiO_2__PHBV nanocomposite is characterized by higher hydrophilic properties, which corresponds to the lower contact angle values than those of pure PHBV. By nature, TiO_2_ nanoparticles tend to accumulate water on their surface, which is why 3 wt.% of nanoparticles in this case induced higher wettability of the nanocomposite. The 1_nTiO_2__PHBV, 5_nTiO_2__PHBV, and 7_nTiO_2__PHBV nanocomposites showed higher water contact angle values, probably due to more crystalline structures (Table 3), which can not absorb water as easily as the amorphous structure. Furthermore, 1 wt.% of nanoparticles was probably too small a portion for the nanocomposite to accumulate water [34]. When talking about contact angles between formamide drops and nanocomposites’ surfaces, a lower value was obtained for the 1_nTiO_2__PHBV nanocomposite, in comparison with the pure PHBV matrix, while the contact angle values for all the rest of the nanocomposites are higher. All of the nanocomposites show similar or stronger hydrophobic properties than the pure PHBV.

The obtained contact angle values were used for surface free energy calculations. In that matter, two models were used: the Owens, Went, Rabel, and Kaelble (OWRK) model and the Wu model. Calculated surface free energy values are shown in Table 7. Generally speaking, the results obtained by these models are somewhat different from one another, since the OWRK model uses the geometric mean value, while the Wu model uses the harmonic mean value of the surface free energy components for calculation. Both models provided a decreasing trend of surface free energy values with an increase in TiO_2_ amount. The existence of the polar and disperse components of surface free energy can be observed in all of the samples, with somewhat higher values of the disperse component. The polar component of the surface free energy represents the hydrophilic nature of the samples, while the disperse component represents their hydrophobic nature [55]. The reason for the higher presence of the dispersed component is the existence of many ethyl and methyl groups in the PHBV molecule structure, which are hydrophobic by nature. The hydrophilic character of the PHBV/TiO_2_ nanocomposite can be attributed to the TiO_2_ nanoparticles [56]. The compact structure of the nanocomposite reduces surface energy, and a lower surface free energy value results in a higher contact angle of the nanocomposite with water and formamide and a lower contact angle with diiodomethane, indicating that the samples are hydrophobic. This property seems to be an advantage for the application of PHBV/TiO_2_ nanocomposites in food packaging production, as microbes would not be able to adhere to hydrophobic surfaces. The material’s low surface free energy also reduces moisture absorption and gas permeability, which is crucial for preserving food quality [57].

### 3.6. Barrier Properties

**Figure 8:**
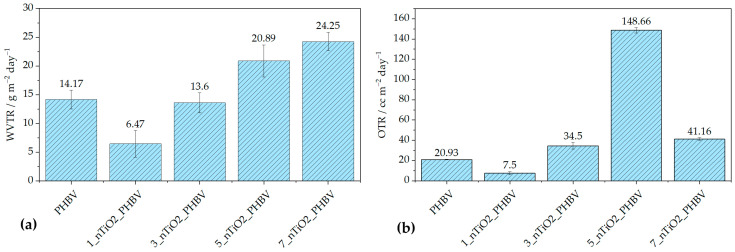
Results of (**a**) WVTR and (**b**) OTR measurements of the pure PHBV and PHBV/TiO_2_ nanocomposites.

The barrier properties of the nanocomposites produced were evaluated using the water vapor transmission rate (WVTR) and oxygen transmission rate (OTR) measurements, which are crucial for assessing the suitability of materials for food packaging. Effective packaging must minimize gas and moisture transmission to maintain product quality and shelf life. Excessive water vapor permeability can create a favorable environment for microbial growth, while for some moisture-sensitive foods, a moderate level of breathability can help maintain freshness and texture. On the other hand, while in contact with oxygen, food can go through a spontaneous oxidation process, which can alter the taste of the food and ultimately the food’s quality. The results of the WVTR measurement are expressed in g m^−2^ day^−1^ (the volume in cubic centimeters of gas passing through one square meter of material per day), and OTR measurements are in cc m^−2^ day^−1^. Both are shown in Figure 8.

Figure 8a describes WVTR behavior depending on the amount of nanoparticles incorporated in the polymer matrix. It can be observed that the smallest portion of 1 wt.% of TiO_2_ induced the improvement of barrier properties with a WVTR value of 6.47 g m^−2^ day^−1^, in comparison with the pure PHBV WVTR value of 14.17 g m^−2^ day^−1^. This can be attributed to the better dispersion of the filler in the matrix, which ultimately caused the formation of more uniform spherulites during the crystallization process. Thus, the free volume of the matrix and polymer chain mobility have been reduced, creating the tortuous, maze-like structure, making it more difficult for water molecules to diffuse through [41,59]. According to Akin and Tihminlioglu (2018) [59], the elongation of the nanoparticles due to the nanocomposite’s processing, as well as their orientation in the matrix, greatly affects the tortuosity of the matrix. Generally speaking, the mentioned literature reports an improvement in water vapor barrier properties with the addition of nanoclay (organic modified montmorillonite) into the PHBV matrix. The results showed a gradual decrease in water vapor permeability with the addition of 1 and 2 wt.% of nanoclay, while larger portions of 3 and 5 wt.% increased it [59]. In other research, like Malmir et al. (2017) [60], PHBV/cellulose nanocrystal (CNC) nanocomposites were prepared. The WVTR tests confirmed the improvement in barrier properties; however, a further increase in CNC loading started to disrupt the nanocomposite structure, thus increasing the WVTR values. Testing of water vapor barrier properties of nanocomposites with TiO_2_ nanoparticles incorporated in the biopolymer matrix has been reported by Venezia et al. (2023) [41] and Brugnoli et al. (2025) [61]. Venezia et al. (2023) [41], as mentioned above, prepared PHBV/TiO_2_/humic acid and reported a decrease in water vapor permeability due to TiO_2_ nanoparticles acting as a physical obstacle for water molecules to pass through. On the other hand, Brugnoli et al. (2025) [29] prepared polyhydroxybutyrate-*co*-hydroxyhexanoate (PHBH) nanocomposites with 1, 2, and 3 phr TiO_2_. WVTR testing showed a drastic and gradual decrease in barrier properties with an increase in the TiO_2_ portion in comparison with pure PHBH. A similar observation was obtained in this research: a gradual increase in WVTR values with the increased TiO_2_ portion. The highest WVTR value was obtained for the 7_nTiO_2__PHBV sample, and it was 24.25 g m^−2^ day^−1^. However, the difference is an improvement in barrier properties with the addition of 1 and 3 wt.% TiO_2_, while higher portions increase the WVTR values. The reason for this kind of behavior lies in the creation of microvoids and defects caused by a higher concentration of nanoparticles, facilitating the diffusion of water vapor [62]. However, these WVTR results are somewhat comparable to the increased wettability of the nanocomposites. Based on the results in Table 6, all of the nanocomposites, except 3_nTiO_2__PHBV, show increased water contact angle, indicating the higher surface wettability. The reason for this is mostly the hydrophilic nature of TiO_2_ nanoparticles, as well as the crystallinity and uniformity of the nanocomposites’ structures. Upon that, a higher concentration of nanoparticles adsorbs water molecules more easily, whilst at the same time, a disrupted structure of the matrix creates more passages for water to diffuse through, thus increasing the WVTR [62].

OTR measurements showed a somewhat similar trend, with one grand exception. In Figure 8b, it can be observed that nanocomposite 1_nTiO_2__PHBV increased the barrier properties towards oxygen, by lowering OTR from 20.93 cc m^−2^ day^−1^ to 7.50 cc m^−2^ day^−1^. The reasons for that are the same as the ones for a decrease in WVTR [59]. Generally, it can be observed that OTR values are higher (except for the nanocomposite with 1 wt.% TiO_2_) than WVTR values. Oxygen molecules are smaller than water molecules, which makes them able to pass more easily through the polymer matrix, especially if it is not uniformly crystallized, as explained in the previous paragraph. Furthermore, the oxygen molecule is of a non-polar nature, which makes it less adsorbable or non-adsorbable by the TiO_2_ nanoparticles; however, it is soluble in the non-polar PHBV matrix [63]. This statement can be confirmed by the increase in the diiodomethane contact angle values on the nanocomposites’ surfaces, as presented in Table 7. In general, the values are lower than the values of contact angles of polar solvents. That means the matrix is, by nature, more non-polar. It can be seen that the diiodomethane contact angle of 5_nTiO_2__PHBV is the highest (52.3 ± 1.9°), indicating the lowest surface free energy value of 41.7 mJ m^−2^ and decreased non-polar nature of the nanocomposite. Nevertheless, this nanocomposite showed the highest OTR value among others (148.66 cc m^−2^ day^−1^). This means that the structure was highly disrupted, with more defects that allowed the passage of the oxygen molecules. This structure was ultimately confirmed and explained by TGA analysis, i.e., the biggest *T_onset_* drop to 260.0 °C (Table 4). Microporosity of the structure, i.e., occurrence of defects on the microscale and poor interfacial adhesion, could also be an explanation [44].

Other studies showed only decreased or maintained values of OTR, in comparison with the pure polymer matrix. Venezia et al. (2023) [41] showed that the addition of TiO_2_ and humic acid to the PHBV matrix did not affect the oxygen permeability much, increasing it from 0.38 ± 0.05 m^3^ m m^−2^ Pa^−1^ s^−1^ (pure PHBV) to 0.40 ± 0.08 m^3^ m m^−2^ Pa^−1^ s^−1^. Such a small change can be attributed to experimental error. Not much literature explains the OTR behavior in PHBV/TiO_2_ nanocomposites. On the other hand, PHBV nanocomposites with organic modified clays, such as montmorillonite, showed differing results. Carli et al. (2016) [39] demonstrated that high concentrations of montmorillonite and halloysite nanotubes cause nanoparticles to agglomerate. Thus, the free volume of the matrix is increased, and barrier properties to oxygen worsen. Based on the SEM results of this research, TiO_2_ nanoparticles did not agglomerate, which is why this explanation is not suitable for the behavior observed. Malmir et al. (2017) [60] observed a large decrease in OTR in PHBV/CNC nanocomposites, in comparison with the pure PHBV. However, the compatibility of the hydroxyl group present in the CNC structure should be taken into account, while in this research, low interfacial adhesion between TiO_2_ and PHBV was confirmed.

### 3.7. Mechanical Properties of the Nanocomposites

**Figure 9:**
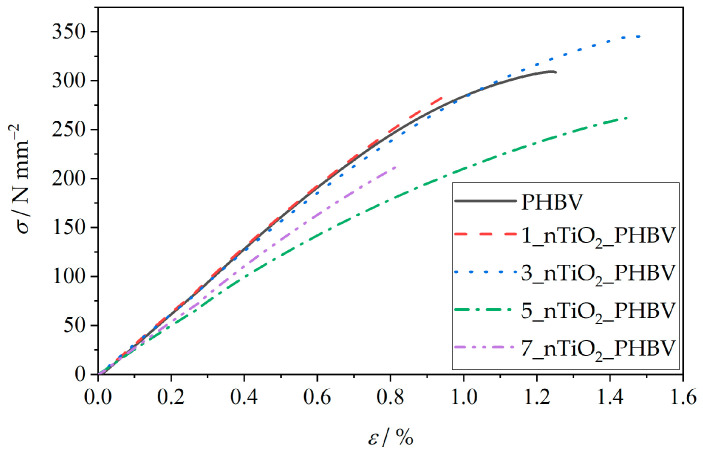
Stress–strain curves of the pure PHBV and PHBV/TiO_2_ nanocomposites.

The packaging must protect the food from any physical damage, which may happen during the food’s transport, storage, or general handling. By understanding the mechanical properties, the producers can ensure that the food packaging will maintain its integrity up until the food’s expiration date. Mechanical properties testing includes sample deformation under the influence of mechanical strain. The results are mostly presented as a curve, which shows the dependence of the strain on the induced stress (stress–strain curve). Results of the stress at break, *σ*, elongation at break, *ε*, and Young’s modulus, *E*, values can be obtained from the same curve. Testing of the mechanical properties is extremely important in the food packaging industry for several reasons. The stress–strain curve discloses the material behavior when it is exposed to mechanical stress. The mechanical stress is defined as the applied force and the material’s cross-sectional area ratio, while strain represents the ratio of elongation and the original length of the sample. The stress–strain curve can be divided into several characteristic stages. As visible in Figure 9, elastic deformation is represented by the linear parts of the curves, at which point the material is able to return to its original state once the applied stress is removed. The yield point is located at the beginning of the curve’s deviation from the linear stage, marking the starting point of permanent deformation. The curve ends with a fracture point, at which stress at break and elongation at break can be obtained. The curve for the PHBV matrix in Figure 9 shows a clearly defined elastic region, followed by a yield point, after which a fracture of the samples can be observed. Based on that, it can be concluded that PHBV is a mostly brittle and fragile material [64]. Since the curves of all of the prepared nanocomposites are of a similar shape, with several exceptions of different *σ*, *ε*, and *E* values, it can be concluded that the nanocomposites have similar mechanical properties.

**Figure 10:**
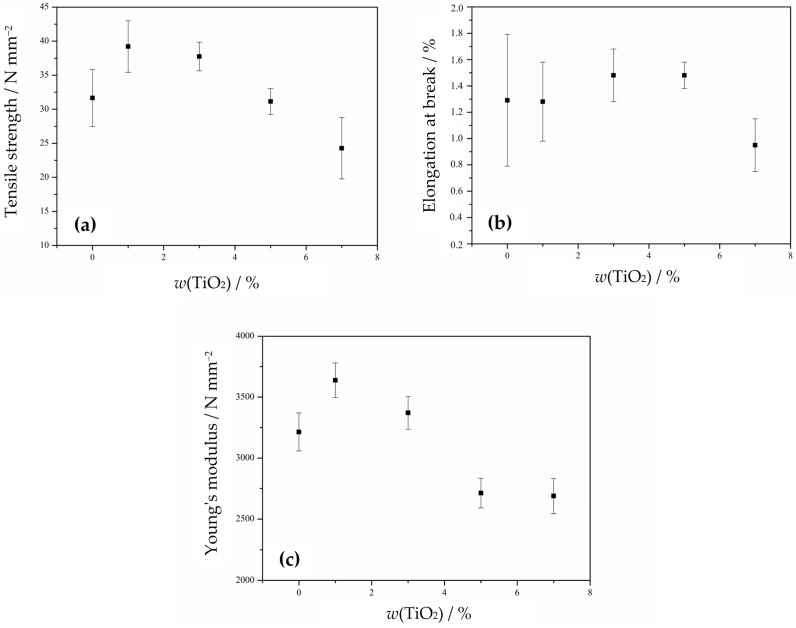
Mechanical properties of the PHBV/TiO_2_ nanocomposites: (**a**) tensile strength; (**b**) elongation at break; (**c**) Young’s modulus.

For a better overview of the mechanical properties, characteristic values of *σ*, *ε*, and *E* are shown in correspondence with the TiO_2_ portion in the nanocomposites in Figure 10. The tensile strength of the neat PHBV matrix totals 300 N mm^−2^ (300 MPa) and represents the maximum amount of stress the PHBV can withstand before breaking. The Young’s modulus of the pure PHBV is approximately 3200 N mm^−2^ (3.2 GPa) and refers to the stiffness of the material, with higher values indicating a stiffer material. The elongation at break value of PHBV ranges from 0.8% to 1.8%. It mostly corresponds to the amount of hydroxyvalerate units in the chain structure, with higher ductility associated with a greater amount of hydroxyvalerate in the PHBV [65].

The addition and increase in TiO_2_ content leads to a decrease in stress at break, although the nanocomposites 1_nTiO_2__PHBV and 3_nTiO_2__PHBV show higher stress at break values compared to the neat PHBV (Figure 10a). The tensile strength then decreases with TiO_2_ contents of 5 and 7 wt.%. The elongation at break values are nearly the same for the PHBV matrix and the 1_nTiO_2__PHBV nanocomposite. Those values furthermore increase with an increase of TiO_2_ to 3 and 5 wt.%, and decrease again after reaching 7 wt.% of nanofillers (Figure 10b). The highest Young’s modulus is observed for the 1_nTiO_2__PHBV nanocomposite, while further increases in TiO_2_ content lead to a decrease. Higher values of Young’s modulus compared to the pure PHBV are shown by the 1_nTiO_2__PHBV and 3_nTiO_2__PHBV nanocomposites (Figure 10c). The nanocomposites 1_nTiO_2__PHBV and 3_nTiO_2__PHBV, compared to the neat PHBV, show higher values of stress at break and Young’s modulus, indicating better mechanical properties of these nanocomposites than the pure PHBV. The elongation at break values showed to be very low (ca. 0.9–1.5%), which could be correlated with a high crystallinity degree (Table 3). High crystallinity causes the material to be brittle and easy to break. Also, as mentioned before, the brittleness is connected with the amount of valerate in the polymer chain structure, which means that more valerate would make the matrix more ductile, and elongation at break would be increased [65]. The obtained results suggest that TiO_2_ nanoparticles are homogeneously dispersed within the polymer matrix, which has been confirmed by SEM analysis before (Figure 4). The improved mechanical properties of these nanocomposites can also be linked to efficient mechanical energy transfer from the nanoparticles and strong filler–matrix interactions [41]. Further increases in TiO_2_ content decrease stress at break, Young’s modulus, and elongation at break values, which indicates more severe mechanical properties in nanocomposites with higher TiO_2_ content. The mechanical properties may also be correlated with the crystallinity degree of the samples, based on DSC analysis results (Table 3). A higher crystallinity improves the tensile strength of the material, as crystalline regions are more densely packed and held together by stronger intermolecular forces. The obtained results, showing higher stress at break values in the 1_nTiO_2__PHBV and 3_nTiO_2__PHBV nanocomposites, are supported by their higher crystallinity (65.6% and 64.8%, respectively) compared to the pure PHBV matrix. However, it would be expected for 5_nTiO_2__PHBV and 7_nTiO_2__PHBV nanocomposites to show greater tensile strength, both 67.6%. Since the DSC analysis results of melting temperature were explained by the general disruption of the structure with higher portions of TiO_2_, the explanation could transfer to the tensile strength as well. The tensile strength values might be lower because of the disorderly packed structure of those nanocomposites, which makes them break under lower stress applied. The available literature provided by Venezia et al. (2023) [41] and Zare et al. (2019) [46] also describes the connection of mechanical properties with the distribution of nanoparticles within the polymer matrix. Based on their conclusions, homogeneous dispersion is key to achieving satisfactory mechanical properties.

### 3.8. Rheological Properties of PHBV and PHBV/TiO_2_ Nanocomposites

**Figure 11:**
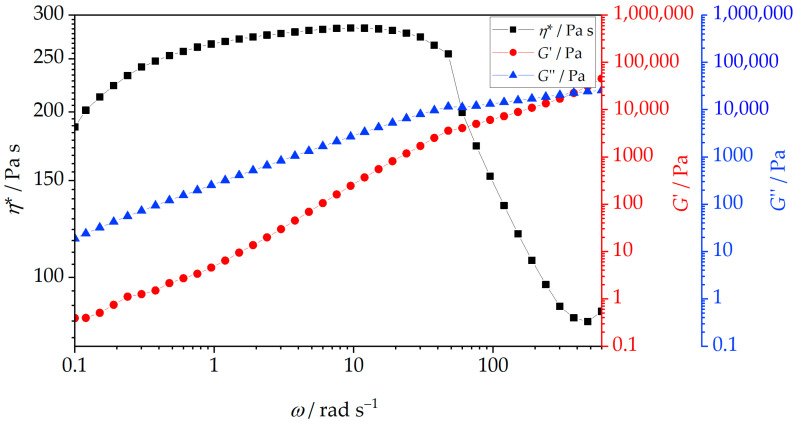
Complex viscosity, η*, storage modulus, *G*’, and loss modulus, *G*″, of PHBV at 180 °C under 2% of dynamic torque strain.

Rheology describes the deformation and flow behavior of materials under applied stress and shear rate. While often associated with liquids, rheological analysis applies to a broad range of substances, many of which exhibit both solid and liquid characteristics, known as viscoelasticity. These materials display both viscous and elastic responses (viscoelastic response), and some of the common examples of them include biological tissues, amorphous polymers, and biopolymers at elevated temperatures. Understanding rheological behavior, particularly viscosity, shear stress, and shear rate, is critical for optimizing the processing of polymer melts and their nanocomposites [66]. The presence, shape, size, and concentration of nanofillers influence the flow properties and structure of nanocomposites, affecting nanofiller dispersion and processing behavior. Figure 11 shows the complex viscosity (η*), storage modulus (*G*′), and loss modulus (*G*″) of neat PHBV as a function of angular frequency. The data indicate a characteristic rheological response of a semicrystalline polyester melt with a relatively fragile viscoelastic network. The complex viscosity is high at low frequencies applied due to strong intermolecular forces and a moderately entangled amorphous phase (e.g., van der Waals interactions), resulting in a solid-like, viscoelastic response. This behavior is visible on the complex viscosity curve (Figure 11) in the low-frequency region, where a broad plateau-like region is observed. The presence of this plateau indicates that, within this frequency range, the relaxation time of the polymer chains is long compared to the applied deformation period, allowing the molecular network to resist flow. With increasing frequency, the complex viscosity decreases markedly, indicating that the molecular network can no longer respond predominantly in a viscous manner to rapid oscillatory deformation, and that the elastic contribution to the viscoelastic response becomes increasingly dominant. In this regime, the melt flows more easily, and the viscous resistance to deformation is reduced. Previous studies [67,68] on the same commercial PHBV (Enmat Y1000P) at 175 °C and 2% strain showed similar behavior of complex viscosity, with a plateau-like region at low frequencies followed by a decrease in viscosity at higher frequencies, which is consistent with the present results. *G*′ (elastic, solid-like behavior) and *G*″ (viscous, liquid-like behavior) further describe the viscoelastic nature of PHBV. In neat PHBV, *G*″ slightly exceeds *G*′ at low frequencies, indicating a dominance of viscous behavior and confirming the liquid-like character of the melt under slow deformation. With increasing frequency, G′ surpasses G″, confirming that the elastic contribution becomes dominant at shorter timescales of deformation. This viscous dominance reflects the good flowability of the melt and the fact that energy dissipation processes outweigh elastic energy storage at low frequencies. With increasing frequency, a crossover between G′ and G″ is observed, marking the transition from viscous-dominated to elastic-dominated viscoelastic behavior. At higher frequencies, the elastic component of the polymer response prevails, indicating that polymer chains are no longer able to fully relax within one oscillation cycle. The predominance of elastic behavior at high frequencies suggests that PHBV does not form a strongly interconnected or highly entangled molecular network in the melt state. Aydemir and Gardner [69] reported similar rheological trends in neat PHB and PHB-based nanocomposites, where viscosity decreased with frequency. Although PHBV was not the polymer investigated in that study [69], the similarity between the rheological behavior of PHB and PHBV can be attributed to their close structural resemblance, while the presence of valerate units in PHBV likely accounts for the observed differences in viscosity. Overall, the rheological behavior of neat PHBV indicates that, although the material exhibits measurable viscoelasticity, its amorphous phase is characterized by a modest entanglement density and limited molecular cohesion in the melt state.

**Figure 12:**
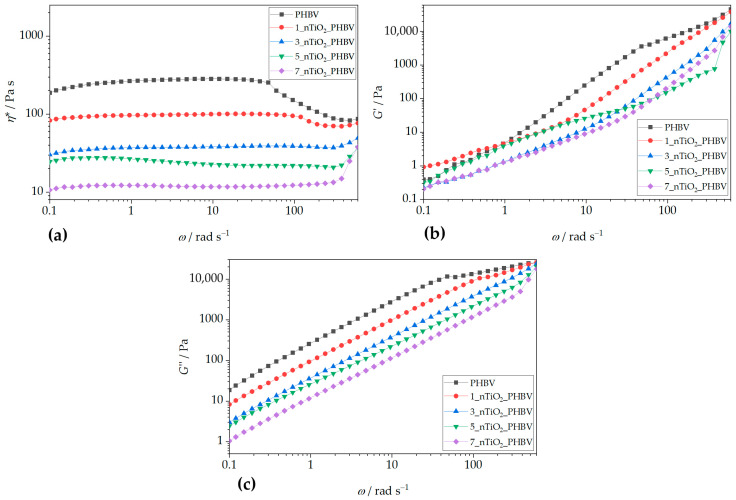
Rheology parameters: (**a**) complex viscosity, η*; (**b**) storage modulus, G’; and (**c**) loss modulus, G″, of pure PHBV and PHBV/TiO_2_ nanocomposites at 180 °C under 2% of dynamic torque strain.

The incorporation of TiO_2_ alters PHBV’s rheological properties (Figure 12). Increasing TiO_2_ content from 1% to 7 wt.% reduces the complex viscosity at higher frequencies (Figure 12a). The 1_nTiO_2__PHBV nanocomposite also exhibits a plateau region, indicating the maintenance of polymer chain coordination and longer relaxation periods. In contrast, 3_nTiO_2__PHBV exhibited a broad plateau at almost all frequencies. Only minor variations in complex viscosity are observed over most of the investigated frequency range, while a noticeable change appears at the highest frequencies, indicating an increased contribution of the viscous component. This phenomenon is also observed in PLA/ZnO composites, prepared by Murariu et al. (2011) [70]. Similar occurrences can be observed for nanocomposites with higher TiO_2_ concentrations (5% and 7 wt.%). Overall, it can be seen that the addition of TiO_2_ in gradually higher concentrations lowers the complex viscosity along all the frequencies applied. The lower concentration of TiO_2_ (1 wt.%) may interact moderately with the polymer matrix, which is why the viscosity drops at higher concentrations. However, more nanoparticles might interfere with chains and thus reduce the effective entanglement in the amorphous phase. Another explanation could be the weak interface interaction between polymer chains and nanoparticles, which act as inert inclusions and interrupt chain–chain interactions. Also, storage and loss moduli both increase rapidly with an increased frequency [71]. A study by Agbakoba et al. (2023) [72] found similar behavior of the PLA and CNCs bionanocomposites. Complex viscosity values were almost the same at lower frequencies applied; however, at higher frequencies, the viscosity increased drastically. This increase in viscosity at higher frequencies is generally undesirable in polymer processing due to increased friction and potential structural damage, making lower nanofiller concentrations (e.g., 1_nTiO_2__PHBV and 3_nTiO_2__PHBV) more favorable for applications like biodegradable food packaging. Contrary findings in the literature [73] reported viscosity increases with TiO_2_ addition, attributed to enhanced polymer chain entanglement. This suggests differences in crystalline structure or nucleation effects in the present study, where lower viscosity may result from TiO_2_-induced disentanglement. Figure 12b,c compare the storage and loss moduli of neat PHBV and TiO_2_-reinforced nanocomposites. Generally, both moduli decrease with increasing TiO_2_ content. At low frequencies, *G*″ > *G*′ for neat PHBV and 1_nTiO_2__PHBV, indicating viscous dominance. At higher frequencies, *G*′ exceeds *G*″, showing elastic behavior, consistent with decreasing η*. Other nanocomposites (3%, 5%, and 7 wt.% TiO_2_) exhibit predominantly viscous behavior (*G*″ > *G*′), further supporting a transition from solid-like to liquid-like behavior with increasing nanoparticle content. This behavior suggests that the presence of TiO_2_ nanoparticles suppresses the development of an elastic network by disturbing polymer–polymer interactions and reducing the effective entanglement density in the amorphous phase. The addition of TiO_2_ progressively reduces the complex viscosity and suppresses the development of an elastic network, especially at higher filler contents. The absence of a clear G′—G″ crossover indicates that TiO_2_ particles do not form a continuous, load-bearing filler network, but rather act as dispersed particles that disturb polymer–polymer interactions and reduce the effective entanglement density. This behavior is beneficial for melt processing and highlights the importance of filler dispersion and interfacial interactions in controlling the viscoelastic response of PHBV/TiO_2_ nanocomposites. From a processing perspective, higher viscosity and indications of weak network formation at the highest loadings can reduce drawability and heat-seal consistency and increase the risk of flow-related defects if dispersion is not well controlled. Accordingly, the rheology results are interpreted as diagnostics of processability (melt strength, film blowing/casting window), whereas packaging suitability is determined by mechanical and barrier outcomes.

### 3.9. Biodegradable Properties

**Figure 13:**
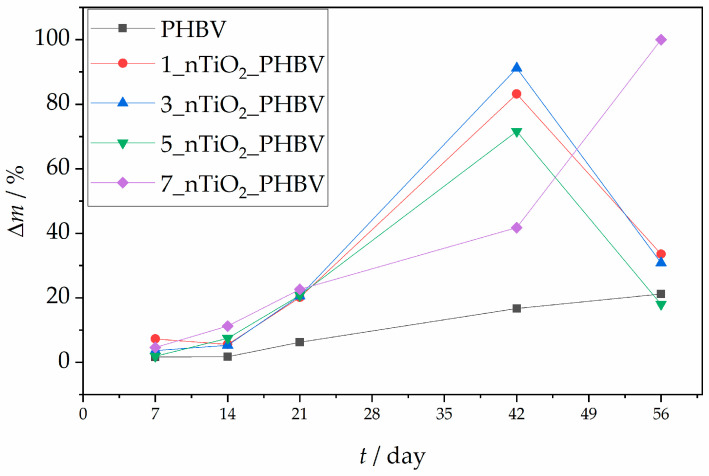
Mass change rates of the pure PHBV and PHBV/TiO_2_ nanocomposites during the biodegradation process.

Biodegradation is a process in which any organic compound is broken down into smaller fragments or molecules by microorganisms. Based on the material’s structure and composition, its biodegradation in the environment can last from days to a few centuries. For example, biodegradable polymers are usually decomposed within six months or a year, whereas the biodegradation process of materials such as synthetic plastic can last much longer. However, the microbes present in the environment have various abilities to metabolize different materials, due to various enzymes they produce, which also affect the time of the biodegradation process [74]. The biodegradation process can occur in environmental conditions with or without the presence of oxygen, which is why aerobic and anaerobic biodegradation processes can be differentiated, respectively. In aerobic processes, the final products of the microbes’ metabolism are primarily carbon dioxide, CO_2_, and water. On the other hand, without any oxygen involved in the process, organic materials are converted into carbon dioxide, water, and methane, CH_4_. Other parameters that affect the degradation process of the materials in the environment are temperature, ultraviolet radiation, moisture, etc. [75]. When talking about polymers, the biodegradation process occurs in four characteristic steps. The crucial step is microorganisms’ attachment and their colonization on the surface. This is the step that initiates the biodegradation process and is followed by biofilm formation due to microorganisms reproducing and growing. Within the biofilm, the enzymes produced by microorganisms break down the polymer into smaller fragments and simpler compounds. This usually occurs due to the presence of the enzyme depolymerase. Furthermore, the polymer chain fractioning into smaller molecules leads to mineralization, i.e., production of CO_2_, water, and/or CH_4_ through the aerobic or anaerobic process [74]. PHBV, which is used in this research, belongs to the biodegradable polymers group. Since it is produced by microorganisms as a reserve energy source by consuming substrates with excess carbon and a lack of nitrogen and other elements, it is easily consumed back by the microbes, which makes it biodegradable. Even if found accumulated outside of their cells, microbes can use it as a carbon source, whilst it is worth mentioning that PHBV’s biodegradability in this manner highly depends on the valerate portion in its structure [76]. Research on this phenomenon was conducted by Mergaert et al. (1992) [77], and the results confirmed an increase in biodegradability with an increase in the valerate portion to 10% and 20%. However, the degradation rate was highly dependent on the temperature used in the experiment, as well as the microorganisms used for the experiment [77]. The possible reason for the increase lies in the decrease in crystallinity, which allows the enzymes and water to break down the amorphous parts of the polymers [78]. Except for the already mentioned depolymerase enzyme, there are other enzymes, such as hydrolases, lipases, peroxidases, and cutinases, which can also be used by microbes to break down PHBV. The specific type of enzyme and its activity can be influenced by the microbial species, the physical properties of PHBV, and some other environmental conditions [74]. In that matter, it takes several weeks or months for it to fully decompose in nature. Titanium dioxide, TiO_2_, which is also used in this research to create nanocomposites with PHBV, is not considered biodegradable because of its inorganic nature. However, it is biocompatible, and it can change and enhance some of the polymer’s properties, which makes the polymer usable in different aspects of life [79]. In that matter, the biodegradable properties of pure PHBV and PHBV/TiO_2_ nanocomposites are assessed in this research. Figure 13 represents the biodegradation process results of pure PHBV and its nanocomposites in soil. In this assessment, the change in mass of the samples was tracked through 7, 14, 21, 42, and 56 days. Based on the results, the mass difference of the pure PHBV was slowly increased during the period of 56 days, with a maximum mass change of 21.21%. The sample was not fully degraded, which corresponds to the literature [79], which states that it takes several months for PHBV to fully degrade by microbial metabolism. Reay et al. [75] conducted a similar experiment in which PHBV microplastics were biodegraded in soil. After 8 weeks, i.e., 56 days, only 1.5 to 5% of the samples were biodegraded. This is due to nitrogen limitation in the substrate (PHBV), which is crucial to initiate microbial growth. Since PHBV does not contain any nitrogen in its molecular structure, microorganisms present in the soil could not grow and enhance their number in the present environment and consume the PHBV. However, the microorganism genera present in the soil also play a significant role in the biodegradation rate, and they were not mentioned in the literature [75]. The decreased biodegradation rate in this research could also be explained by the nitrogen limitation. All of the nanocomposites tested showed a higher rate of mass loss; however, the results were highly varied. The highest mass difference on the 56th day was observed for the 7_nTiO_2__PHBV nanocomposite. This nanocomposite was fully biodegraded after 56 days, while other nanocomposites showed lower mass differences after 56 days, in comparison with their mass difference obtained after 42 days. The reason for the full biodegradation of the 7_nTiO_2__PHBV nanocomposite lies in its structure, which benefits the biodegradation. As mentioned before, due to the larger nanoparticle content, the PHBV matrix structure becomes more disrupted, with unsystematically formed crystals, and more continuous and exposed amorphous regions [41]. This was explained by the lowered melting temperature obtained from DSC analysis (Table 3). This means that this nanocomposite had a more amorphous structure, which is more beneficial for microbes to consume and degrade the polymer [80]. Thus, the higher mass loss was observed. On the other hand, nanocomposites 1_nTiO_2__PHBV, 3_nTiO_2__PHBV, and 5_nTiO_2__PHBV showed higher rates of mass loss up to the 42nd day of the biodegradation process; however, the mass difference was lowered on the 56th day for all three of the nanocomposites. Since all of the amorphous regions of the PHBV matrix were consumed by the microorganisms, the TiO_2_ nanoparticles and the crystalline regions of the PHBV matrix emerged on the surface of the nanocomposites. Since both nanoparticles and crystalline regions are susceptible to biodegradation, they started to adsorb water molecules present in the soil, thus enhancing the mass of the remaining sample and lowering the final mass difference [80]. Moreover, the results of WVP present in Figure 10 confirm that these nanocomposites have lower barrier properties towards water vapor, which means that water molecules can penetrate into the nanocomposites’ structure more easily. Similar results were obtained in research by Zare et al. (2019) [46] by testing PHBV–chitosan nanocomposites with integrated ZnO and Ag nanoparticles. The enhancement in mass of the samples was observed after 8 weeks of biodegradation, which was attributed to the water adsorption on the nanocomposite’s surface [46]. Similarly, Braga et al. (2018) [32] conducted biodegradation testing of PHBV/TiO_2_ nanocomposites with 1, 2.5, and 5 wt.% nanoparticles for 20 days. The biodegradation process generally resulted in a weight loss exceeding 60%; the highest weight loss (~82%) was observed for the nanocomposite containing 2 wt.% TiO_2_. However, previous studies have concluded that TiO_2_ nanoparticles tend to reduce the rate of biodegradation compared to pure PHBV, which contrasts with the findings of the present study [32].

**Figure 14:**
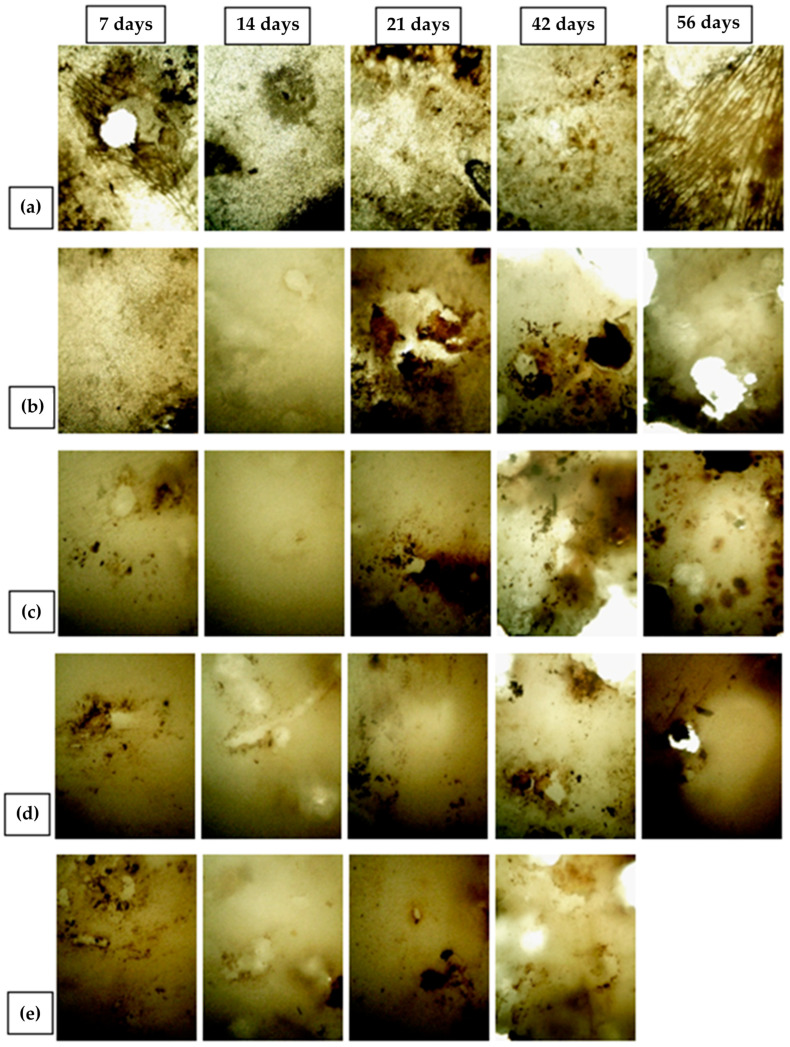
Microphotographs (optical microscope) of (**a**) pure PHBV; (**b**) 1_nTiO_2__PHBV; (**c**) 3_nTiO_2__PHBV; (**d**) 5_nTiO_2__PHBV; (**e**) 7_nTiO_2__PHBV nanocomposite, after 7, 14, 21, 46, and 56 days of biodegradation process.

**Figure 15:**
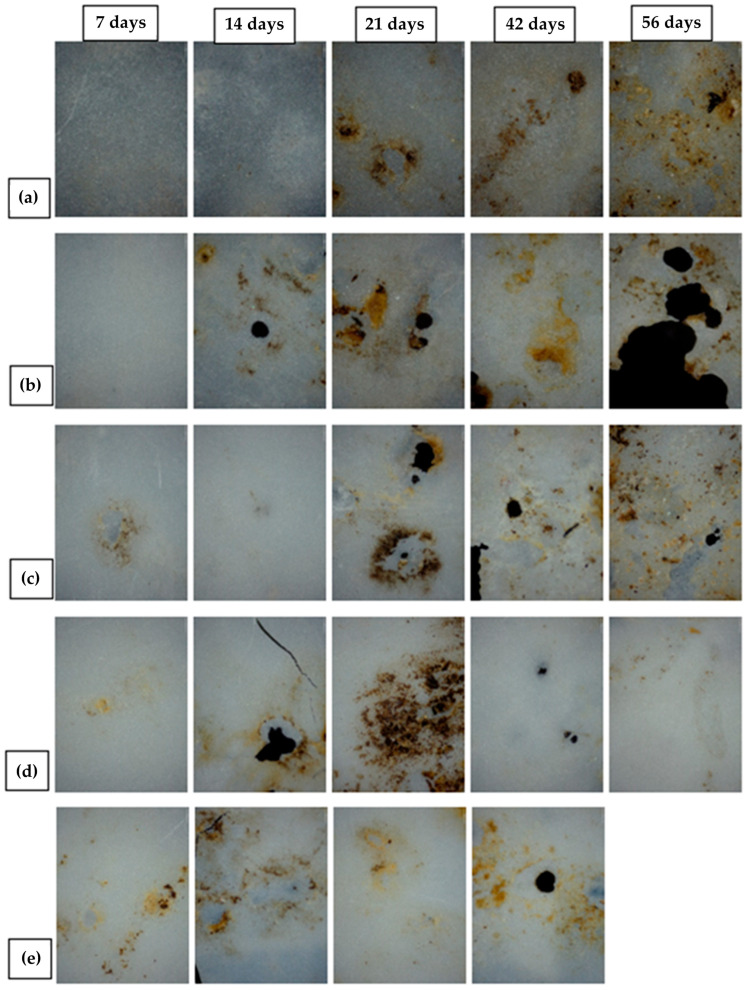
Microphotographs (polarizing microscope) of (**a**) pure PHBV; (**b**) 1_nTiO_2__PHBV; (**c**) 3_nTiO_2__PHBV; (**d**) 5_nTiO_2__PHBV; (**e**) 7_nTiO_2__PHBV nanocomposite, after 7, 14, 21, 46, and 56 days of biodegradation process.

Figure 14 and Figure 15 present microphotographs of PHBV and its nanocomposites, captured using optical and polarizing microscopy, respectively. Both figures illustrate the surface macrostructure of the samples after 7, 14, 21, 42, and 56 days of biodegradation. A progressive degradation of the sample surfaces is evident over time, including the formation of holes attributed to microbial activity. Notably, microphotographs for day 56 are absent in Figure 14e and Figure 15e, as the corresponding sample was fully degraded, as previously discussed and shown in Figure 13. In most of the micrographs in Figure 14, numerous black and white spots are visible, which correspond to bacterial colonies. Their presence confirms microbial colonization and indicates that biodegradation indeed occurred. In Figure 14a, representing pure PHBV on day 56, dark linear structures can be seen, which are identified as fungal hyphae. This suggests that moulds also contributed to the biodegradation process, consistent with their ability to grow on the polymer surface [81]. Compared to the PHBV/TiO_2_ nanocomposites shown in Figure 14b–e, the surface of the pure PHBV sample exhibited more extensive microbial colonization and was more consistently covered with microbial biofilms throughout the experiment. In contrast, the surfaces of the nanocomposites appeared smoother and showed fewer dark spots, indicating a lower rate of microbial colonization. However, by days 42 and 56, the nanocomposites displayed a greater number of holes relative to pure PHBV, suggesting more pronounced degradation. These observations are consistent with the measured mass loss, particularly for the 7_nTiO_2__PHBV sample, which exhibited complete degradation (100% mass loss). The polarizing micrographs (Figure 15) provide additional insight into the morphological changes in the samples, highlighting structural disruption caused by microbial activity. Residual brown and yellow regions visible on the surfaces represent adherent soil particles and biofilms that could not be completely removed. The quantity of these residues appears to increase over time, likely influenced by surface roughness. While the amount of residue on nanocomposites is comparable to that on pure PHBV, the nanocomposite samples exhibited more pronounced surface perforation after 42 and 56 days of exposure.

**Figure 16:**
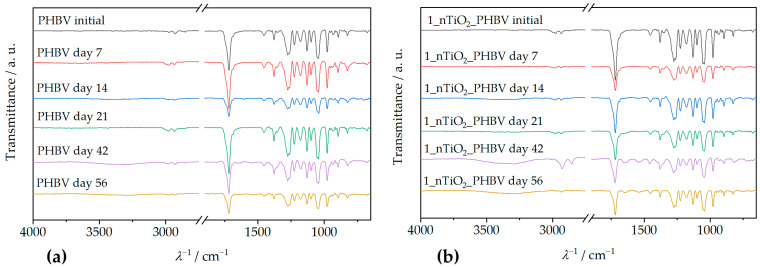

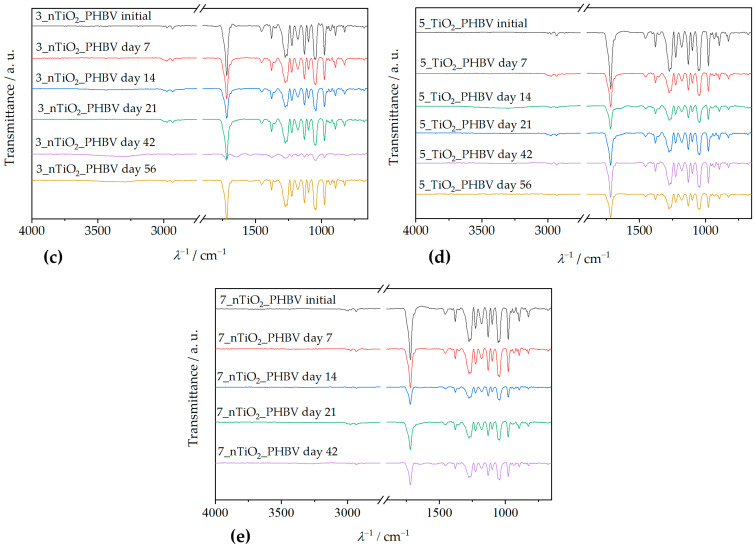
FTIR spectra obtained before (initial) and after 7, 14, 21, 42, and 56 days of biodegradation of (**a**) the pure PHBV; (**b**) 1_nTiO_2__PHBV; (**c**) 3_nTiO_2__PHBV; (**d**) 5_nTiO_2__PHBV; (**e**) 7_nTiO_2__PHBV.

FTIR analysis of all of the samples was conducted in order to track their biodegradation. Figure 16 represents FTIR spectra of the samples of pure PHBV and 1_nTiO_2__PHBV, 3_nTiO_2__PHBV, 5_nTiO_2__PHBV, and 7_nTiO_2__PHBV during the biodegradation process, respectively. All of the spectra show the peaks of O–H bond stretching, C–H bond stretching and bending, as well as C=O and C–O bond stretching. Wavenumbers at which these peaks occur are shown in Table 5. Figure 16a shows FTIR spectra of the pure PHBV sample throughout the biodegradation process. As stated before, all of the spectra show the characteristic peaks of the PHBV structure. In comparison with the initial FTIR spectrum, as the days of the biodegradation process pass, a slight shift of the carbonyl bond stretching peak towards higher wavenumber values can be observed, which corresponds to the microbial hydrolysis of the esteric bond [82]. This occurrence indicates that the biodegradation process really happened. Spectra obtained for 1_nTiO_2__PHBV (Figure 16b) show a slightly broader shift of the same bond peak towards higher wavenumber values, as the time of the experiment passes. This means that biodegradation occurred as well; however, the 1_nTiO_2__PHBV were slightly more degraded than the pure PHBV. On the same spectrum, a broader and sharper peak for O–H bond stretching emerges on the 42nd and 56th days. This corresponds to the previously given conclusions for the lower mass difference at the end of the biodegradation process, which state that the adsorption of the water molecules occurred. As stated before, water was adsorbed on the samples by the end of the biodegradation process, and that can be confirmed with FTIR spectra as the O–H bond stretching peak becomes more and more pronounced as time passes. Thus, the O–H bond stretching peaks can be seen on the FTIR spectra. Similar results were obtained for the nanocomposites 3_nTiO_2__PHBV and 5_nTiO_2__PHBV, where the shift of the carbonyl bond stretching peak happened after the determined time periods. Also, on both of the spectra in Figure 16c,d, O–H bond stretching peaks can be observed, which are getting sharper and broader with time due to the adsorption of water. The FTIR spectrum of 7_nTiO_2__PHBV in Figure 16e shows the biggest shift in the carbonyl bond stretching peak in comparison with the initial peak. This confirms the highest percentage of mass loss, i.e., the highest biodegradation occurrence among all the samples tested. It can also be seen on the spectrum that the O–H bond stretching peaks do not exist on any of the spectra obtained after the specific periods of the experiment, which means that water did not adsorb on the polymer surface. This also corresponds to the fact that the mass loss is higher and higher as time passes, as visible in Figure 16.

## 4. Conclusions

In this study, PHBV/TiO_2_ nanocomposites were synthesized and systematically evaluated for their thermal, mechanical, morphological, barrier, and biodegradation properties, with neat PHBV serving as the reference material. The DSC analysis shows that TiO_2_ incorporation subtly modifies the melting and crystallization behavior of PHBV, primarily through nucleation effects rather than large structural changes. TGA results show that TiO_2_ generally enhances the thermal stability of PHBV by increasing *T_onset_* and *T_max_*, although this effect is not strictly linear with filler content due to interfacial defects at higher loadings. These properties are attributed to the effective dispersion of TiO_2_ nanoparticles within the PHBV matrix, as confirmed by SEM/EDX imaging and supported by rheological data. Furthermore, the barrier tests suggest that low TiO_2_ loadings improve barrier properties against oxygen and water vapor, due to the occurrence of a more tortuous structure. Notably, while the 7 wt.% TiO_2_ nanocomposite (7_nTiO_2__PHBV) showed some deviations in property trends; it also exhibited the highest biodegradation rate under microbial conditions. This suggests that, despite minor structural inhomogeneities, 7_nTiO_2__PHBV holds the greatest environmental promise, whereas lower quantities of the TiO_2_ nanoparticles ensure better properties for sustainable food packaging needs. However, such changes in the properties are not packaging-relevant, as the nanocomposites did not deliver a robust, multi-property improvement sufficient to justify adoption for food-packaging applications. Thus, general mechanical properties improvement, and further investigation on the nanoparticles’ surface treatment, migration to food, alternative fillers, etc., are obligatory in order to develop an optimal material for this application.

## Figures and Tables

**Table 1:** Specification of PHBV matrix.

Property	Value
Melt flow index (g (10 min)^−1^)	5–15
Tensile strength at break (N mm^−2^)	39
Elongation at break (%)	2
Young’s modulus (N mm^−2^)	2.800–3.500
Flexural modulus (N mm^−2^)	3.520–4.170
Melting point (°C)	170–176

**Table 2:** Composition of PHBV/TiO_2_ nanocomposite samples.

Sample ID	*w* (TiO_2_)/%	*m* (PHBV)/g	*m* (TiO_2_)/g
PHBV	0	40.0	0.0
1_nTiO_2__PHBV	1	39.6	0.4
3_nTiO_2__PHBV	3	38.8	1.2
5_nTiO_2__PHBV	5	38.0	2.0
7_nTiO_2__PHBV	7	37.2	2.8

**Table 3:** Results of pure PHBV and PHBV/TiO_2_ nanocomposite DSC analysis.

Samples	*T*_m_/°C	Δ*H*_m_/J g^−1^	*T*_c_/°C	Δ*H*_c_/J g^−1^	*χ_c_*/%
PHBV	171.8	92.0	124.8	87.5	63.0
1_nTiO_2__PHBV	172.0	95.8	125.9	89.2	66.3
3_nTiO_2__PHBV	171.1	94.6	126.0	86.1	66.8
5_nTiO_2__PHBV	168.7	93.8	125.8	86.8	67.6
7_nTiO_2__PHBV	166.7	91.8	125.4	84.2	67.6

**Table 4:** Results of thermogravimetric analysis for pure PHBV and PHBV/nTiO_2_ nanocomposites.

Samples	TG				DTG
	*T*_onset_/°C	*T*_f_/°C	Δ*m*/%	*R*_700°C_/%	*T*_max_/°C
PHBV	265.0	310.2	98.3	1.1	294.8
1_nTiO_2__PHBV	283.0	301.9	98.6	1.3	297.2
3_nTiO_2__PHBV	284.0	302.9	96.8	2.8	297.1
5_nTiO_2__PHBV	260.0	292.3	95.1	4.6	285.3
7_nTiO_2__PHBV	277.4	301.3	93.5	6.2	295.3

**Table 5:** Wavenumber values obtained from the FTIR spectra of the PHBV/TiO_2_ nanocomposites.

Vibrations	Samples			
	1_nTiO_2__PHBV	3_nTiO_2__PHBV	5_nTiO_2__PHBV	7_nTiO_2__PHBV
C-H bending	1356	1380	1380	1380
	1380	1404	1404	1402
	1453	1453	1453	1453
C-H stretching	2854	2850	2850	2846
	2929	2934	2933	2934
	2977	2978	2981	2977
	2997	2993	-	2997
C=O bending	1718	1718	1718	1718
C-O bending	1261	1261	1262	1262
	1275	1273	1275	1275
C-O-C bending	1053	1055	1055	1054
	1100	1100	1100	1100
	1131	1130	1130	1130
	1180	1181	1181	1181
	1227	1226	1227	1226
Ti-O-Ti bending	658	678	674	677

**Table 6:** Contact angle values obtained from the testing of the pure PHBV and PHBV/TiO_2_ nanocomposites with polar and non-polar liquids.

Samples	Contact Angle, *θ (°)*		
	Polar Liquids		Non-Polar Liquids
	Water	Formamide	Diiodomethane
PHBV	67.8 ± 1.1	54.8 ± 0.4	41.1 ± 1.2
1_nTiO_2__PHBV	70.8 ± 1.9	54.1 ± 2.0	41.6 ± 2.4
3_nTiO_2__PHBV	67.0 ± 1.4	57.9 ± 0.8	47.0 ± 0.7
5_nTiO_2__PHBV	72.7 ± 1.9	61.6 ± 0.8	52.3 ± 1.9
7_nTiO_2__PHBV	68.9 ± 1.2	65.1 ± 2.6	44.2 ± 2.2

**Table 7:** Disperse components, polar components, and surface free energy values of the pure PHBV and PHBV/TiO_2_ nanocomposites.

Samples	Surface Free Energy/mJ m^−2^					
	OWRK Model			Wu Model		
	*γ^d^*	*γ^p^*	*γ*	*γ^d^*	*γ^p^*	*γ*
PHBV	34.7	8.9	43.6	34.6	12.5	47.1
1_nTiO_2__PHBV	35.5	7.5	43.0	35.2	11.3	46.5
3_nTiO_2__PHBV	31.4	10.3	42.0	31.8	13.4	45.3
5_nTiO_2__PHBV	29.4	8.4	37.8	30.3	11.4	41.7
7_nTiO_2__PHBV	30.8	9.0	39.8	31.8	11.6	43.4

## Data Availability

The raw data supporting the conclusions of this article will be made available by the authors on request.
